# A bibliometric analysis of studies on artificial intelligence in neuroscience

**DOI:** 10.3389/fneur.2025.1474484

**Published:** 2025-01-27

**Authors:** Ugur Tekin, Murat Dener

**Affiliations:** ^1^Department of Information Security Engineering, Graduate School of Natural and Applied Sciences, Gazi University, Ankara, Türkiye; ^2^Neuroscience and Neurotechnology Center of Excellence (NÖROM), Gazi University, Ankara, Türkiye

**Keywords:** neuroscience, artificial intelligence, bibliometric analysis, scientometric analysis, research trend

## Abstract

The incorporation of artificial intelligence (AI) into neuroscience has the potential to significantly enhance our comprehension of brain function and facilitate more effective diagnosis and treatment of neurological disorders. Artificial intelligence (AI) techniques, particularly deep learning and machine learning, offer transformative solutions by improving the analysis of complex neural data, facilitating early diagnosis, and enabling personalized treatment approaches. A bibliometric analysis is a method that employs quantitative techniques for the examination of scientific literature, with the objective of identifying trends in research, evaluating the impact of influential studies, and mapping the networks of collaboration. In light of the accelerated growth and interdisciplinary scope of AI applications in neuroscience, a bibliometric analysis is vital for mapping the landscape, identifying pivotal contributions, and underscoring emerging areas of interest. This study aims to address this need by examining 1,208 studies published between 1983 and 2024 from the Web of Science database. The analysis reveals a notable surge in publications since the mid-2010s, with substantial advancements in neurological imaging, brain-computer interfaces (BCI), and the diagnosis and treatment of neurological diseases. The analysis underscores the pioneering role of countries such as the United States, China, and the United Kingdom in this field and highlights the prevalence of international collaboration. This study offers a comprehensive overview of the current state and future directions of AI applications in neuroscience, as well as an examination of the transformative potential of AI in advancing neurological research and healthcare. It is recommended that future research address the ethical issues, data privacy concerns, and interpretability of AI models in order to fully capitalize on the benefits of AI in neuroscience.

## 1 Introduction

The fields of artificial intelligence (AI) and neuroscience have become two important and complementary disciplines that have developed rapidly in recent years. Artificial intelligence, particularly with the application of deep learning and machine learning techniques, offers transformative solutions in the comprehension of brain functions and in the diagnosis and treatment of neurological diseases (1).

A bibliometric analysis provides a quantitative assessment of scientific literature using various metrics and is an important tool for identifying trends, key topics, and influential studies in a particular research area (2). A bibliometric analysis of studies in neuroscience and AI can reveal research trends and potential future research areas at the intersection of these two disciplines.

Furthermore, the role of AI in the diagnosis and treatment of neurological diseases is also becoming more prominent. In the early diagnosis of neurological disorders such as Alzheimer's disease, Parkinson's disease, and epilepsy, AI has demonstrated promising results with high accuracy rates (3). Furthermore, the utilization of AI in the formulation of bespoke treatment plans for the management of these conditions is on the rise (4).

Interdisciplinary studies integrating two critical disciplines, neuroscience and artificial intelligence, have elucidated solutions to numerous significant problems at both theoretical and applied levels. These studies have yielded a plethora of innovative solutions, ranging from a more profound comprehension of brain mechanisms to the development of artificial intelligence models. However, given the nascent state of the field, it is evident that further comprehensive and in-depth research is imperative, with a focus on the synergies between neuroscience and artificial intelligence. It is imperative to underscore that this research is not merely a desirable objective, but rather an urgent necessity, given the rapid advancements in technology.

The present study will conduct a comprehensive bibliometric analysis of the literature on the applications of artificial intelligence (AI) in neuroscience. Additionally, it will examine research trends at the intersection of these two disciplines in great detail.

This article presents a bibliometric analysis of artificial intelligence studies in neuroscience. By presenting a comprehensive analysis of the scientific literature published in this field,

- publication trends,
- publication-citation-author relations,
- country-institution collaborations have been revealed.

Using bibliometric analysis techniques,

- shed light on the current state of research in the field of artificial intelligence in neuroscience,
- Identified key areas to focus on,
- sub-field trends in their work in this field over the years.

The remainder of the paper is structured as follows: the Section 2 presents a review of related studies in the literature and outlines the methodology employed in the present study. The Section 3 presents and elucidates the results of the bibliometric analysis. The Section 4 elucidates the significance of the study, analyzes the findings, and reinterprets them in the context of the research question. The Section 5 presents the conclusions and implications of the study.

## 2 Literature review and methodology

### 2.1 Literature review

Bibliometric analyses in the fields of neuroscience and artificial intelligence (AI) have made significant contributions to the identification of research trends and key topics at the intersection of these disciplines. This section will examine similar studies in the literature on the application of artificial intelligence in neuroscience.

In a study published in 2023, Cui et al. (5) investigated the development of artificial intelligence in radiomics toward nervous system diseases. The study demonstrated that AI is a significant contributor to the analysis and interpretation of nervous system diseases with a notable increase in the number of publications in this field.

Esteva et al. (1) conducted a comprehensive analysis of the application of AI in the diagnosis of neurological diseases. The study demonstrated that AI achieved high accuracy rates in the early diagnosis of disorders such as Alzheimer's disease, Parkinson's disease, and epilepsy.

Xia et al. (6) conducted a bibliometric study in neuroscience, with a particular focus on ophthalmology. The objective of this study is to conduct a comprehensive bibliometric analysis within the field of neuroscience, with a particular focus on research related to ophthalmology. The aim is to facilitate the dissemination of knowledge within the scientific community, enabling researchers, practitioners, and policymakers to gain insight into the current state and future potential of neuroscience research focused on ophthalmology.

In a recent study, Lin et al. (7) investigated the trends of neuroscience in major information systems journals. The objective of the study is to conduct a comprehensive bibliometric analysis of neuroscience research published in major information systems journals. The objective of the research is to map the evolution and development of the interdisciplinary field that combines neuroscience and information systems, identify influential works, and ascertain emerging areas of interest.

In a study published in 2022, Wang et al. (8) investigated the potential applications of neuroscience tools in the field of building construction. The objective of the study is to investigate the potential applications of neuroscience tools and methods in the field of building construction. The study aims to provide guidance for future research by identifying promising avenues for further investigation and collaboration between the fields of neuroscience and building construction.

In a study published in 2022, Ali et al. (9) investigated the efficacy of machine learning algorithms in the diagnosis of epilepsy. The study demonstrated that AI exhibits high accuracy in predicting and classifying epileptic seizures. In a related vein, Chen et al. (10) conducted a bibliometric analysis of AI applications in neurology. The study underscores the growing significance of AI in neurological research and the considerable surge in the volume of publications in this domain.

In a study conducted by Yeung et al. (11), the objective of the study is to conduct a comprehensive bibliometric analysis of neuroscience research conducted between the years 2006 and 2015. The objective of the research is to identify the key trends and patterns in neuroscience research that have emerged over the specified period, thereby providing insights into the evolution of the field. The objective of the study is to evaluate the evolution of the focus of neuroscience research over time, identifying emerging areas of interest and changes in research priorities.

In a study published in 2024, Reddy et al. (3) investigated the potential applications of artificial intelligence (AI) in the treatment of Parkinson's disease. The study demonstrated that AI plays a pivotal role in disease monitoring and treatment plan personalization. In a similar vein, Duda et al. (12) examined the effectiveness of neural network models in the diagnosis of autism spectrum disorder. The study demonstrated that AI is a valuable tool for early diagnosis and intervention.

Aslam et al. (13) conducted an analysis of the utilization of AI in the field of multiple sclerosis research. The study demonstrated that AI exhibits high accuracy in predicting disease progression and identifying disease subtypes. In a study published in 2017, Litjens et al. (14) explored the potential of deep learning techniques in the diagnosis of neurodegenerative diseases. Their findings indicated that AI demonstrated considerable promise in the early detection of such diseases.

Despite the numerous studies that have investigated the intersection of neuroscience and artificial intelligence (AI), this research offers a distinctive contribution by providing a comprehensive bibliometric analysis of studies specifically focused on AI applications in neuroscience. In contrast to previous studies, which frequently emphasize specific subfields or applications, our research provides a more comprehensive overview of the broader landscape by identifying trends, influential works, and emerging research areas in this interdisciplinary field.

In summary, our study distinguishes itself by providing the first large-scale bibliometric analysis of AI research in neuroscience, covering various subfields, analyzing historical and emerging trends, and identifying critical areas for future research. This comprehensive approach not only fills gaps in the existing literature but also serves as a roadmap for researchers, practitioners, and policymakers in this rapidly evolving field.

These studies offer a comprehensive overview of research in both neuroscience and AI, elucidating significant findings at the nexus of these two disciplines. Bibliometric analyses offer insights that are invaluable for identifying research trends and shaping future research directions.

### 2.2 Methodology

**Figure 1:**
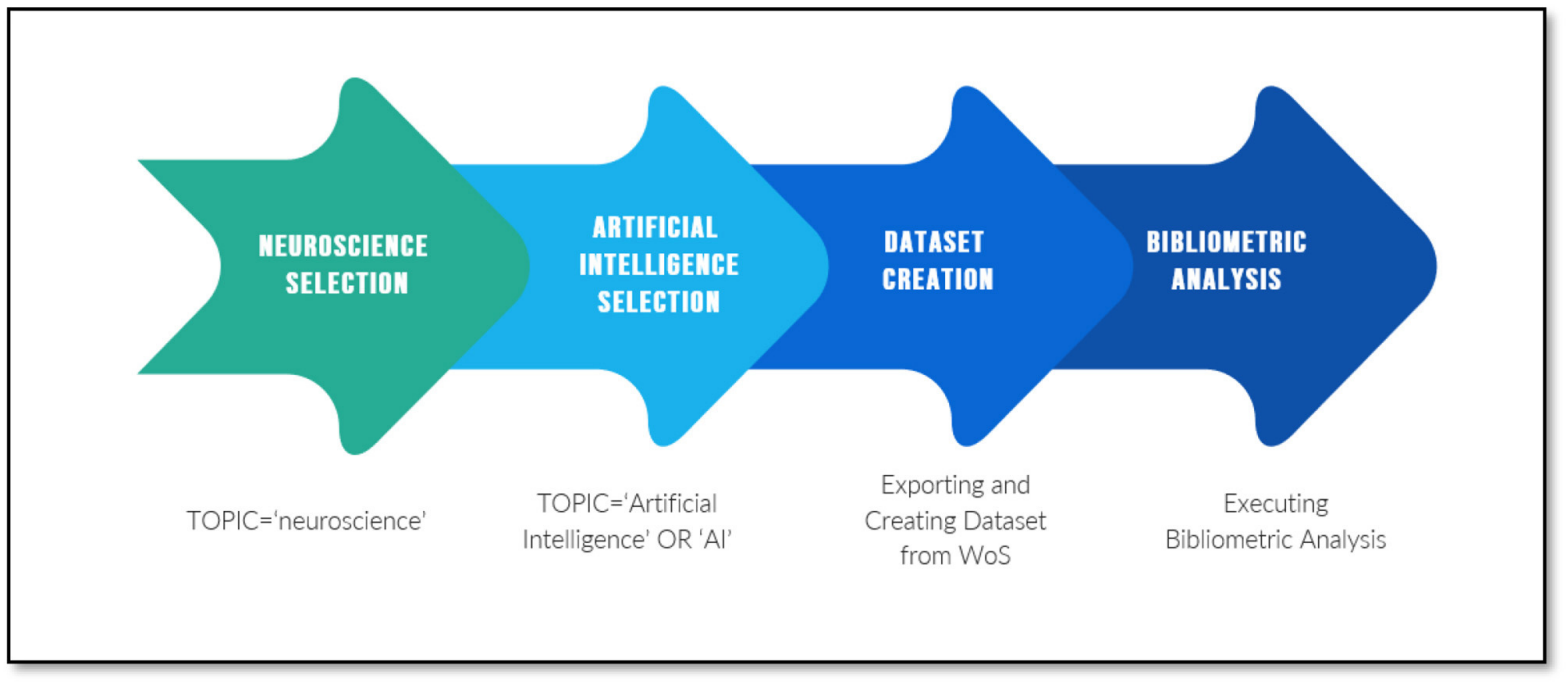
Dataset selection process.

A systematic literature review was conducted using the Web of Science (WoS) database to gain insight into AI studies in neuroscience, to reveal the evolution of the fields of study over the years, and to identify trends in the field. In order to generate a comprehensive list of AI studies in neuroscience, as illustrated in Figure 1, the search topic field was first entered with the terms “TOPIC” = “neuroscience” AND (“Artificial Intelligence” OR “AI”). The resulting list was then subjected to analysis in order to ascertain the evolution of the fields of study over the years and to identify trends within the field.

Consequently, all studies on AI in neuroscience have been cataloged, resulting in a dataset comprising 1,208 studies. The dataset was created on July 3, 2024, and thus, publications published subsequent to this date were not included. The aforementioned dataset was subsequently exported to Web of Science and analyzed with the Bibliometrix and VOSviewer tools. Bibliometrix and VOSviewer are two software programs that are frequently utilized in the realm of bibliometric analysis.

Bibliometrix was developed as an open-source R package, enabling comprehensive analysis of scientific publications. Despite its lack of a user-friendly interface, Bibliometrix is a highly flexible and powerful tool for researchers proficient in R programming. Bibliometrix facilitates analyses such as the examination of annual trends, geographical distributions, keyword networks, and author collaborations within scientific publications. It also offers extensive functionality for advanced bibliometric analyses, such as trend analyses, clustering techniques, and conceptual mapping (2).

VOSviewer is a specialized software that focuses on network visualization and mapping. When used in conjunction with a user-friendly interface, it provides a visual representation of scientific publications, citations, keywords, and relationships between institutions. The software's ability to process large data sets and identify links between thematic clusters is noteworthy. It is particularly well-suited for the creation of conceptual maps, the analysis of author collaboration networks, and the evaluation of journal co-attribution analysis (15).

A notable strength of VOSviewer is its complementarity to other tools, offering features that address the limitations of standalone applications. Bibliometrix provides a robust infrastructure for conducting statistical analyses and metric calculations, while VOSviewer enhances the visualization and interpretability of data. Consequently, many researchers opt to utilize both tools in tandem to comprehensively address the quantitative and visual dimensions of bibliometric analysis.

## 3 Data analysis and results

### 3.1 Overview

**Figure 2:**
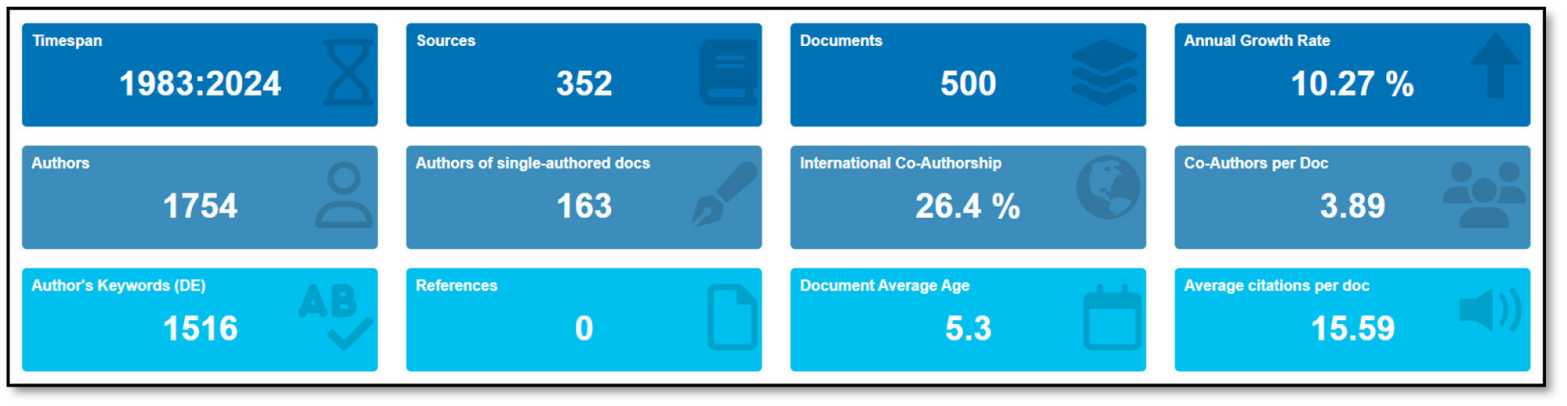
An overview of dataset.

After the creation of the dataset of artificial intelligence applications in neuroscience, the analysis of the data began. An overview of the information contained in the dataset is presented in Figure 2.

The bibliometric analysis of applications of artificial intelligence in neuroscience for the period 1983–2024 shows that research activities in this field are continuously increasing. A total of 1,208 documents from 744 sources were analyzed, with an annual growth rate of 12.32%. This rate reveals a rapidly growing interest in the integration of AI and neuroscience. Among the 4,076 authors participating in the studies, the number of single-authored documents was 305 and the international co-authorship rate was 26.32%. The fact that the number of co-authors per document is 3.96 shows that research in this field is generally carried out in teams. The fact that the number of keywords used by the authors is 3,745 indicates that the research topics cover a wide range. There are a total of 44,773 references in the documents analyzed, reflecting the breadth and depth of the literature. The average number of citations per document is 21.85 and the average age of the documents is 5.43 years, indicating that the studies are both current and influential. These findings underscore that the integration of neuroscience and artificial intelligence is a rapidly evolving, collaborative, and wide-ranging area of research.

**Figure 3:**
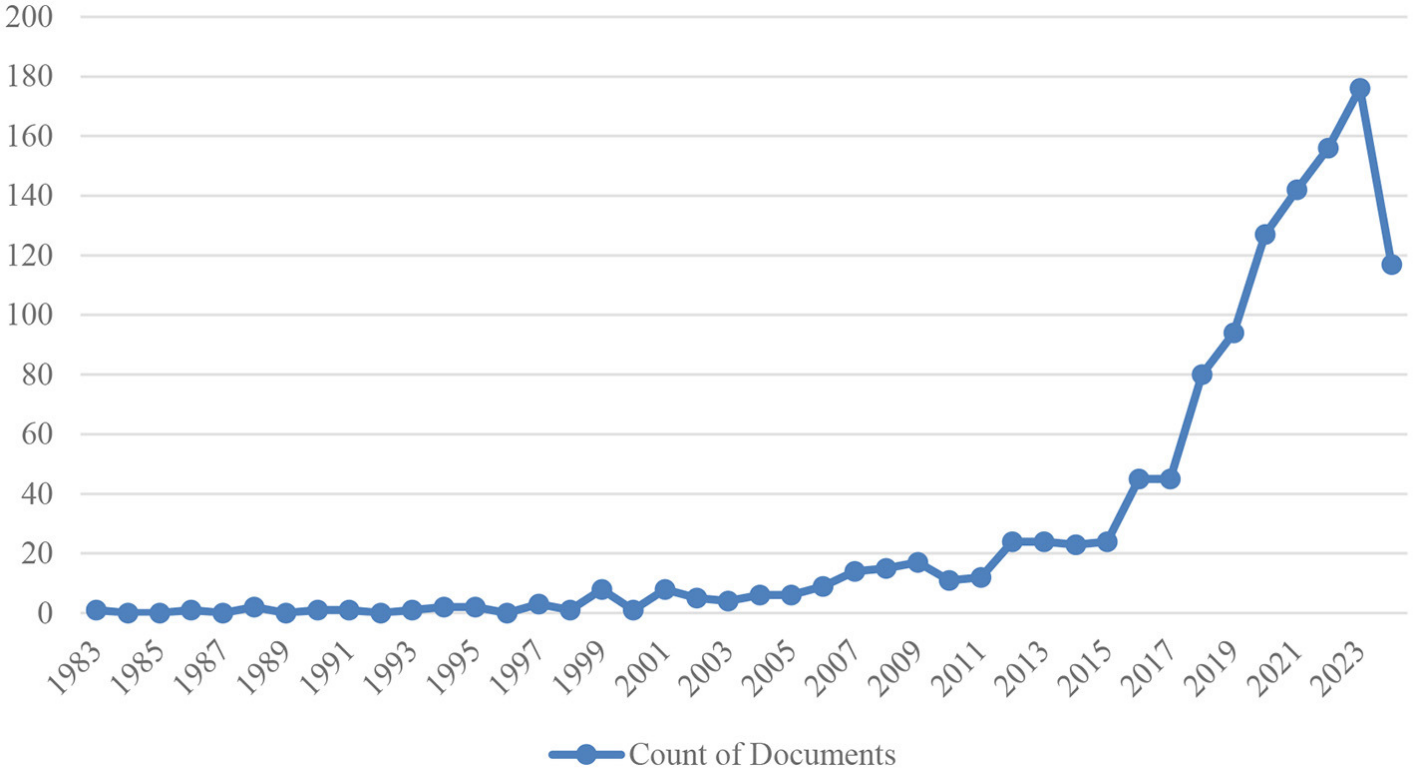
Counts of documents by publication years.

Figure 3 shows the distribution of studies on artificial intelligence applications in neuroscience by year.

From 1983 onwards, research activities in this field started at a very low level and increased minimally until the 1990s. Toward the end of the 1990s (especially in 1999 and 2001) there was a significant increase, with eight papers published in each of these years. This is probably due to the acceleration of technological developments in the fields of neuroscience and artificial intelligence, and the increased interest of researchers in combining these two disciplines.

In the early 2000s (2000–2007), the number of documents published annually varied between 1 and 14. Between 2008 and 2011, this number continued to increase, reaching between 11 and 17 documents. From 2012 onwards, a significant upward trend can be observed. Between 2012 and 2015, the annual number of documents stabilized between 23 and 24, and doubled to 45 in 2016 and 2017.

Since 2018, research on artificial intelligence applications in neuroscience has continued to increase rapidly, reaching 94 documents in 2019, 127 in 2020, 142 in 2021, 156 in 2022 and 176 in 2023. For 2024, 117 documents have been published with the available data, and it is estimated that this number may increase further by the end of the year.

The integration of neuroscience and artificial intelligence has garnered significant attention in recent years, with a marked increase in research endeavors focused on this intersection. The substantial increase in research output can be attributed to several pivotal factors, including technological and clinical elements. Technological advances, including the development of high-performance computing systems, machine learning algorithms, and deep neural networks, have led to substantial increases in the capacity to analyze and interpret large-scale, complex neuroscience datasets. Furthermore, advancements in neuroimaging technologies, such as functional magnetic resonance imaging (fMRI), electroencephalography (EEG), and other brain mapping tools, have facilitated unparalleled access to comprehensive brain data, thereby creating a conducive environment for AI applications.

This field has gained significant momentum since the mid-2010s and has now become a major research focus. This trend is expected to continue, driven by the integration of innovative and effective AI applications in neuroscience, leading to both theoretical advances and practical outcomes. A comprehensive understanding of the technological and clinical dynamics underpinning this growth is imperative to shape the future trajectory of this interdisciplinary field and fully actualize its potential.

### 3.2 Source analysis

There are many sources for publishing AI research in neuroscience, but some of them are more relevant to the field of cybersecurity and attract the attention of their readers with publications in this field. Table 1 provides a bibliometric analysis of the top sources publishing AI articles in neuroscience, including the number of articles and the quartile (*Q*) index, which measures the impact of the journal.

**Table 1:** Most relevant sources (top 10).

**Sources**	**Articles**	**Q Index**
Frontiers in Neuroscience	24	Q2
Frontiers in Computational Neuroscience	19	Q2
Neuron	14	Q1
Cognitive Computation	13	Q2
Neural Networks	12	Q1
Cognitive Systems Research	11	Q3
Nature Communications	11	Q1
Neurocomputing	11	Q1
Brain Sciences	10	Q3
Frontiers in Human Neuroscience	10	Q3

The analysis of the journals with the highest number of publications in the field of AI applications in neuroscience shows that this interdisciplinary work is addressed in a broad spectrum. The journal Frontiers in Neuroscience stands out with 24 articles and is in the Q2 category, indicating that it is followed by a broad academic community. Similarly, Frontiers in Computational Neuroscience ranks second with 19 articles and is in the Q2 category, highlighting the prevalence of computational neuroscience research.

In third place, in the Q1 category, is the highly influential journal Neuron, with 14 articles. The prestige of this journal shows the scientific importance of AI applications in neuroscience and that research in this area is being discussed on high-quality platforms. Cognitive Computation is in the Q2 category with 13 articles, demonstrating the importance of studies that combine neuroscience and artificial intelligence.

Neural Networks, in the Q1 category, highlights the critical role of neural networks and artificial intelligence algorithms in neuroscience with 12 articles. Cognitive Systems Research, in the Q3 category with 11 articles, hosts more niche and specialized studies on cognitive systems. Nature Communications, also in the Q1 category, provides a high-impact platform in this area with 11 articles.

The journal Neurocomputing is also in the Q1 category with 11 articles, highlighting the importance of studies in artificial intelligence and computational neuroscience. The journal Brain Sciences, in the Q3 category, covers various studies in neuroscience with 10 articles, showing that research in this field is of interest to a wide scientific audience. Finally, Frontiers in Human Neuroscience is also in the Q3 category with 10 articles, highlighting the importance of artificial intelligence applications in human neuroscience.

These findings suggest that the integration of neuroscience and artificial intelligence has been widely explored across different sub-disciplines and at different levels of influence, and that research in this area has met with increasing interest.

**Figure 4:**
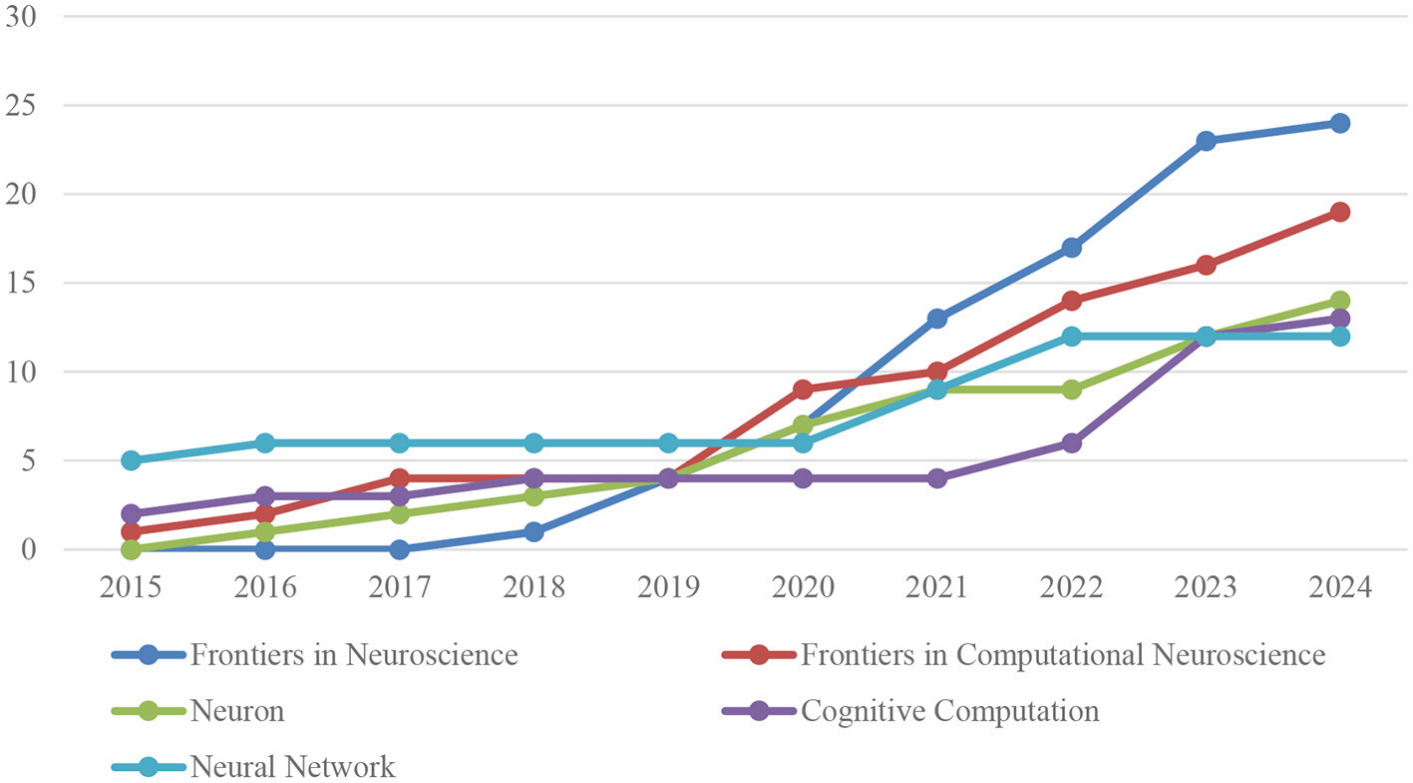
Sources production over time (top 5).

Figure 4 shows the number of publications over time in the Top 5 journals on AI in neuroscience.

A quantitative analysis of the annual publication output of the five journals with the highest number of publications on AI applications in neuroscience reveals a marked increase in research activities in this field in recent years. Since 2015, the change in the annual number of publications in each journal can be summarized as follows.

Frontiers in Neuroscience did not publish any articles on artificial intelligence in neuroscience until 2018, and then entered this field with one article in 2018. This number increased to four articles in 2019, seven articles in 2020, 13 articles in 2021, 17 articles in 2022, 23 articles in 2023 and 24 articles in 2024, showing a significant increase.

The Frontiers in Computational Neuroscience journal published a single article in 2015, but this increased to two articles in 2016. In 2017 and 2018, the number of articles published increased to four, but remained stable in 2019. In 2020, the number of published articles increased significantly, reaching nine. This upward trend continued in 2021, with the publication of 10 articles, and in 2022, 2023, and 2024, with the publication of 14, 16, and 19 articles, respectively.

Prior to 2016, the Neuron journal did not publish any articles in this field. In 2016, one article was published, followed by two in 2017, three in 2018, and four in 2019. In 2020, the journal published seven articles, nine in 2021, and a further nine in 2022. In 2023, the number of articles published increased to 12, and in 2024, this figure rose again to 14.

The journal Cognitive Computation published two articles in 2015, three in 2016, and four in 2017. In 2018 and 2019, the journal demonstrated consistent output with four articles each, and maintained this level of output in 2020. In 2021, four articles were published, and this number increased to six in 2022, 12 in 2023, and 13 in 2024.

The number of articles published in the Neural Networks journal increased from five in 2015 to six in each of the subsequent 4 years, namely 2016, 2017, 2018, and 2019. In 2020, the number of published articles remained at six, but in 2021, there was a notable increase to nine. In 2022, the journal published 12 articles, and in both 2023 and 2024, it maintained a consistent output of 12 articles per year.

The journals Frontiers in Neuroscience and Frontiers in Computational Neuroscience have made substantial contributions to the development of the field, as evidenced by the substantial number of publications and the quality of their content. These journals have also demonstrated a high level of impact. These journals serve as conduits for diverse and high-quality research, catalyzing scientific advancement through the promotion of innovative methodologies and interdisciplinary collaborations. Furthermore, the studies disseminated through these journals serve as a catalyst for both the academic community and applied sciences, fostering an increase in international collaborations among researchers. These distinguishing characteristics position these journals as prominent publications within the domains of neuroscience and computational neuroscience, offering substantial contributions to the advancement of scientific knowledge.

The analysis demonstrates a notable surge in publications on artificial intelligence (AI) applications in neuroscience, particularly since 2018, accompanied by a discernible intensification of research activity in this domain in recent years. Each journal has made a distinct contribution to this field, with an increasing level of activity over time. This increasing trend demonstrates that the integration of neuroscience and artificial intelligence is becoming an increasingly significant area of scientific inquiry, attracting considerable interest from researchers.

### 3.3 Country and affiliation analysis

This section analyses the authors, countries and universities/institutes to which the author is affiliated.

Table 2 presents the top 10 authors and their publication information in the field of artificial intelligence applications in neuroscience. Authors' contributions in this field are evaluated by taking into account their names, total number of publications, fractionalized number of articles and affiliated institutions.

**Table 2:** Most relevant authors (top 10).

**Authors**	**Articles**	**Articles Fractionalized**	**Affiliations**
Wang Y	13	3.09	University of Technology Sydney
Wang X	10	2.10	University of Alberta
Zhang Y	8	1.37	Beijing Jiaotong University
Botvinick M	7	1.21	Hunan University
Chen J	7	1.20	North Carolina State University
Chen X	7	1.14	University of Michigan
Li J	7	1.56	Fujian University of Technology
Li Y	7	2.78	Fujian University of Technology
Liu X	7	0.58	Southeast University
Wang J	7	2.07	Central University of Finance and Economics

The author with the highest number of publications is Wang Y, from the University of Technology Sydney, who has published 13 papers and has a fractionalized paper count of 3.09. This evidence demonstrates that Wang Y has made a substantial contribution to this field of study. Wang X, from the University of Alberta, is in second place with a total of 10 papers and a fractionalized paper count of 2.10, which indicates a significant level of contribution.

Zhang Y, a researcher from Beijing Jiaotong University, has published eight papers, with an average fractionalized number of 1.37. Botvinick M, from Hunan University, has made a notable impact with seven published papers and a fractionalized paper count of 1.21. Chen J, from North Carolina State University, has published the same number of articles, with a fractionalized article count of 1.20. Chen X, from the University of Michigan, has published the same number of articles and has a fractionalized article count of 1.14.

Li J and Li Y are affiliated with Fujian University of Technology and have both published a total of seven papers. However, Li J's fractionalized number of articles is 1.56, while Li Y's fractionalized number of articles is 2.78. This discrepancy suggests that Li Y's impact in this domain is more extensive. Liu X, from Southeast University, published the same number of articles but demonstrated the lowest level of contribution, with a fractionalized article count of 0.58. Ultimately, Wang J, from Central University of Finance and Economics, published seven articles, resulting in a fractionalized number of articles of 2.07.

The universities with which these authors are affiliated also provide information about the academic environments and research infrastructures in which they operate. Notably, Fujian University of Technology has made a substantial impact in this domain, with two authors contributing extensively to the field. The table illustrates that research in the domain of artificial intelligence applications in neuroscience is conducted across a diverse range of disciplines, with select universities demonstrating a particular prominence in this field.

**Figure 5:**
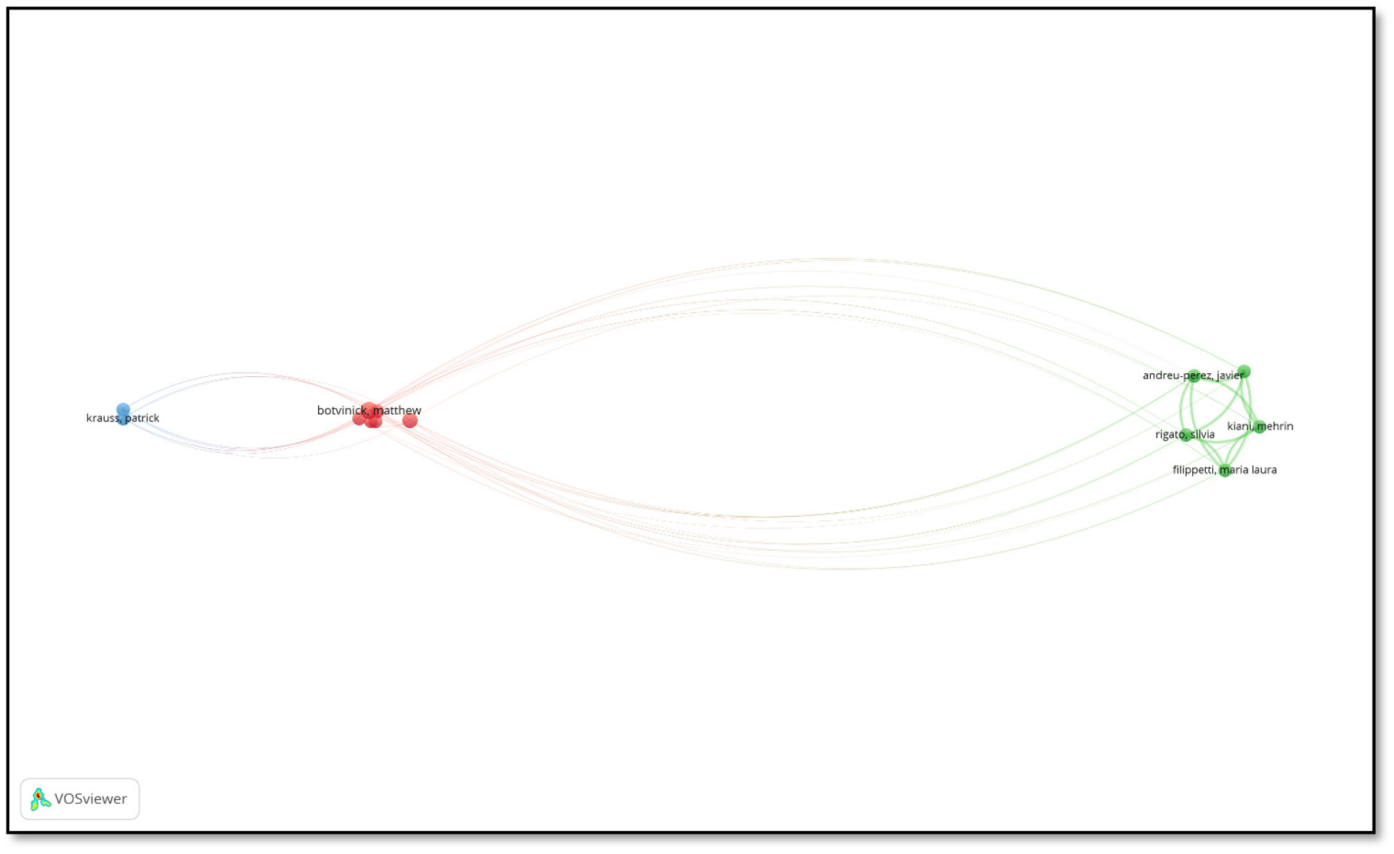
Co-authorship of authors.

Figure 5 is a network map that reveals the co-authorship relationships between authors.

The size of each node represents the author's centrality within the network, as well as the intensity of their collaborative activity. For example, the image illustrates that Maria Laura Filippetti has a large node and a large network of connections, indicating her close collaboration with other authors. The lines indicate the existence of a co-authorship relationship between the authors, while the thickness and density of the lines represent the strength of the collaboration. The analysis depicted in the image illustrates that specific groups of authors engage in more frequent and robust collaboration with one another, thereby forming a cohesive network of connections within their respective groups. Nevertheless, certain authors, such as Hani Hagras, seem to act as conduits between disparate groups, thereby facilitating the growth of the collaborative network. The mapping of scientific collaborations enables the visualization of the social dynamics within the field, the identification of central researchers, and the identification of potential opportunities for collaboration.

Table 3 presents a ranking of the ten universities with the highest number of publications in the field of artificial intelligence applications in neuroscience, indicating the countries of origin and the number of articles published by each institution. The data illustrate the research contributions and academic activities of these universities in this field.

**Table 3:** Most relevant affiliations (top 10).

**Affiliation**	**Country**	**Articles**
Stanford University	U.S.A.	48
University of Oxford	United Kingdom	44
Tsinghua University	China	35
Fudan University	China	33
Harvard University	U.S.A.	33
University of Minnesota	U.S.A.	32
McGill University	Canada	31
University of Chinese Academy of Sciences	China	30
University of Pennsylvania	U.S.A.	28
University of Zurich	Switzerland	28

A total of 48 papers have been published by Stanford University, USA, representing the highest number of publications among all institutions included in this study. This evidence indicates that Stanford is a preeminent institution in the field of research at the nexus of neuroscience and AI. The University of Oxford in the United Kingdom occupies the second position with 44 papers, thereby demonstrating a robust research contribution in this domain.

Tsinghua University from China is in third place with 35 papers, representing a significant contribution to the field from one of China's leading academic institutions. Similarly, Fudan University is another notable contributor from China with 33 papers. Harvard University from the US is in fourth place with the same number of papers (33), which serves to illustrate the university's robust presence in neuroscience and AI research.

The University of Minnesota, USA, has made a noteworthy contribution with 32 articles. McGill University, located in Canada, is a significant research institution in this field, contributing 31 articles to the body of literature. The University of Chinese Academy of Sciences from China is included in this list with 30 articles, which serves to illustrate the extensive research capacity that China has in this field.

The University of Pennsylvania in the United States of America is a significant contributor to this field, with 28 papers published on the topic. With an identical number of publications (28), the University of Zurich in Switzerland is a notable research hub in this field.

**Figure 6:**
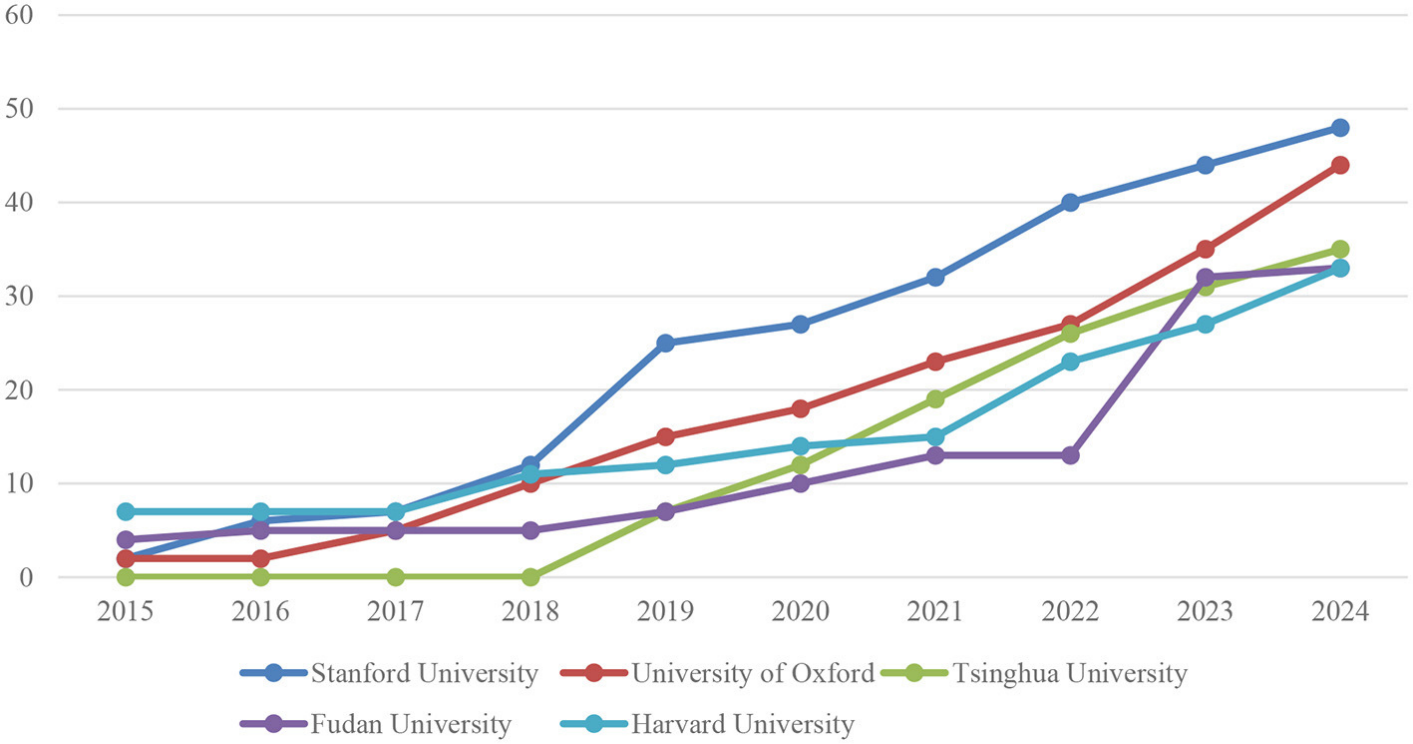
Most relevant affiliations production over time (top 5).

Figure 6 illustrates the total number of publications by year for the five universities with the highest number of publications in the field of artificial intelligence applications in neuroscience. In light of the observed annual growth rates, the following interpretation of the table is warranted:

The number of articles published by Stanford University has demonstrated a consistent upward trajectory, beginning with two articles in 2015 and culminating in 48 articles in 2024. In particular, there was a notable surge in the number of articles published between 2018 and 2019, with an increase from 12 to 25. Subsequently, there was a gradual increase in the number of articles published, reaching a total of 48 articles by 2024. This evidence demonstrates that Stanford's research capacity and contribution in this domain are expanding at a rapid rate.

The University of Oxford commenced with two articles in 2015 and subsequently augmented its output to 44 articles by 2024. The rise in the number of Oxford articles is particularly notable since 2018. The number of articles published by the University of Oxford has exhibited a consistent upward trajectory, beginning with 10 articles in 2018 and reaching 15 articles in 2019, 18 articles in 2020, and 44 articles in 2024.

Tsinghua University did not publish any articles between 2015 and 2017. However, there has been a notable surge in output since 2018. The number of articles published by Tsinghua University increased rapidly from 2018 to 2024. In 2018, the university published no articles. In 2019, it reached a total of seven articles, in 2020, it published 12 articles, and in 2024, it published a total of 35 articles. This demonstrates that, despite commencing its involvement in this field at a relatively late stage, Tsinghua University has begun to make a significant contribution at a rapid pace.

Fudan University commenced with four articles in 2015 and concluded with 33 articles in 2024. Fudan demonstrated a relatively consistent growth between 2015 and 2018, but a more pronounced expansion in the number of articles was observed from 2019 onwards. The number of articles published by Fudan University increased significantly over the course of the study period. In 2019, the university published seven articles, in 2020 it published ten, and in 2024 it published 33.

Harvard University began with seven articles in 2015 and reached a total of 33 articles by 2024. Harvard has consistently produced a relatively high number of articles from its inception and has demonstrated a pattern of steady growth in this output. The upward trajectory commenced with 11 articles in 2018, 12 articles in 2019, 14 articles in 2020, and culminated in 33 articles in 2024, indicative of a sustained expansion.

In general, these five universities are making notable contributions to the field of artificial intelligence (AI) applications in neuroscience. The annual rates of increase in the number of articles in each of the aforementioned institutions demonstrate a clear upward trajectory in research activities and scientific outputs within this field. Notably, Stanford University and the University of Oxford are at the vanguard of this field, exhibiting particularly rapid and sustained growth. Despite its relatively recent involvement in the field, Tsinghua University has demonstrated a notable increase in activity. Additionally, Fudan University and Harvard University merit mention for their consistent and incremental growth. The presented data demonstrate that research in neuroscience and artificial intelligence is expanding at a rapid pace on a global scale, and that these universities are consolidating their leading roles in this field.

**Figure 7:**
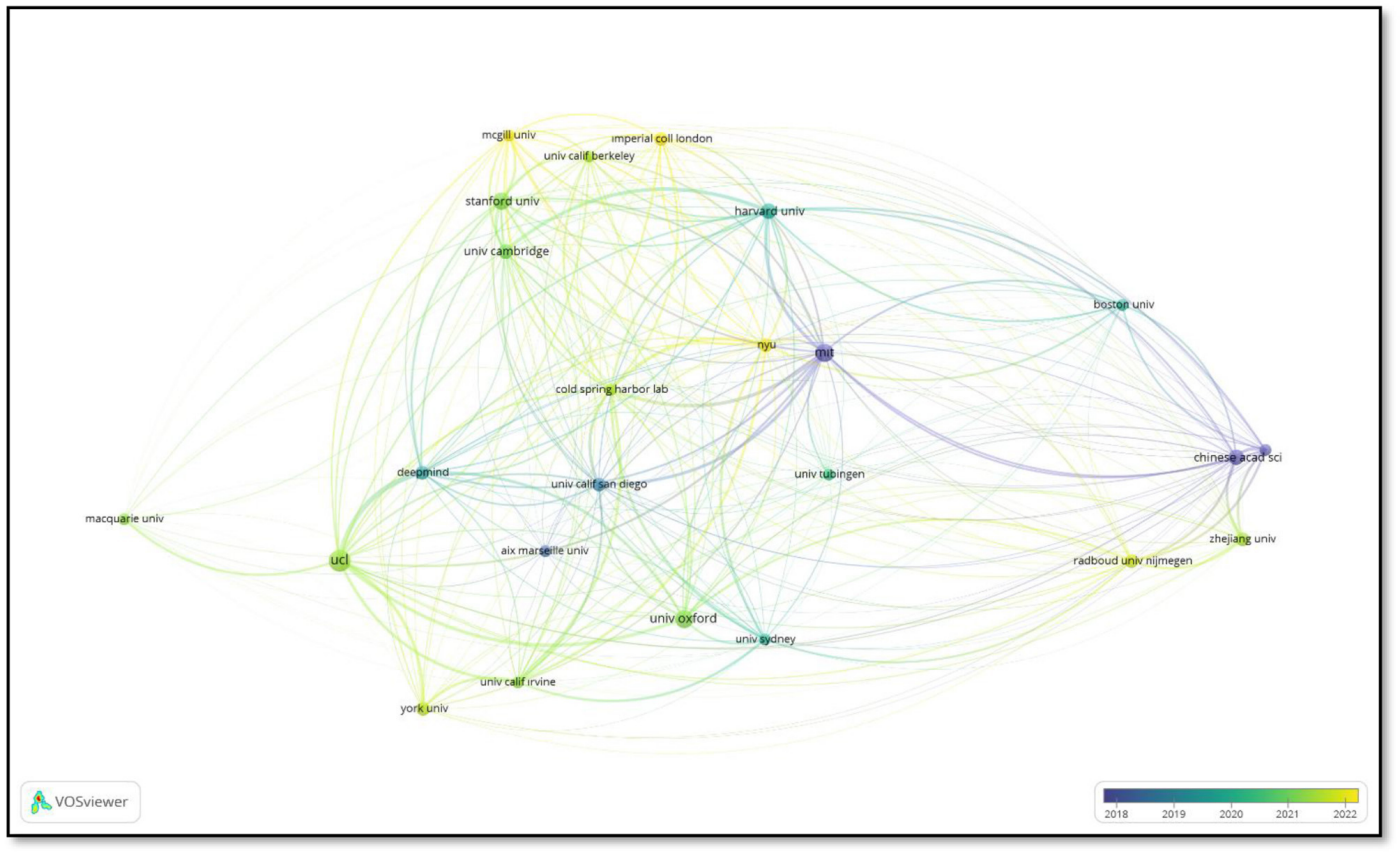
Co-authorship of organizations.

Figure 7 illustrates the network of co-authorship between organizations. The size of each node represents the link density of the corresponding organization in the co-authorship network. The lines represent collaborations between institutions, while the colors indicate different time intervals.

The Massachusetts Institute of Technology (MIT) occupies a pivotal role within the network, having established robust collaborative relationships with numerous other academic institutions. Notably, it has established close affiliations with Harvard University, New York University (NYU), and the University of California, Berkeley (UCB). This illustrates MIT's preeminent position within the scientific collaboration network. Similarly, the Chinese Academy of Sciences occupies a central role in scientific collaborations within East Asia and has a substantial network of global partnerships. University College London (UCL) represents another noteworthy hub within the European co-authorship network, with connections to institutions such as the University of Oxford and DeepMind.

The image also serves to illustrate the geographical diversity and the cooperative relationships that have developed over time. For example, the links in lighter colors demonstrate the increasing intensity of collaborations in recent years. By mapping the scientific interactions between institutions, this analysis provides an important tool for understanding the global distribution and impact of research collaborations.

Table 4 presents a ranking of the 10 countries with the highest number of publications in the field of artificial intelligence (AI) applications in neuroscience, along with their respective total number of articles. The data enables an evaluation of the research contributions and scientific activity of the respective countries in this field.

**Table 4:** Most relevant country (top 10).

**Country**	**Articles**
USA	1,213
China	714
United Kingdom	439
Germany	308
Canada	261
Italy	221
France	205
Spain	185
Japan	148
India	127

The United States of America (USA) has by far the largest number of publications in this field, with a total of 1,213 papers. This shows that the US is a leader in research at the intersection of neuroscience and artificial intelligence and accounts for the majority of scientific activity in this field. The US has an extensive research network and many leading universities and research centers in this field, which is one of the main reasons for this high number of papers.

China ranks second with 714 papers, representing one of its major contributions in this field. China's large investments in scientific research in recent years and its rapidly increasing research activities in neuroscience and artificial intelligence explain this high number of articles. China's rapid rise in this field is remarkable.

The United Kingdom occupies the third position with a total of 439 articles, thereby illustrating its substantial contribution to research in this domain. The United Kingdom is home to numerous universities and research centers engaged in significant neuroscience and artificial intelligence studies, occupying a leading position in this field.

Germany is in fourth place with a total of 308 articles and is one of the major research centers in Europe. Germany places a high value on scientific research, as evidenced by its significant output in this field. Canada ranks fifth with 261 articles and makes notable contributions in neuroscience and artificial intelligence. Universities and research institutions in Canada are engaged in active research in this field, resulting in the production of scientific publications.

Italy occupies the sixth position with a total of 221 articles and is characterized by an active research environment in this field. Italy's engagement with and impact on the fields of neuroscience and AI are reflected in the number of articles published.

France occupies the seventh position with a total of 205 articles and is engaged in significant research activities in the domains of neuroscience and artificial intelligence. France's commitment to scientific research is evidenced by the number of publications in this field.

Spain occupies the eighth position with a total of 185 articles and an active research environment in this field. Spain's contribution to scientific research is evidenced by the number of articles published.

Japan occupies the ninth position with a total of 148 articles and is engaged in substantial research activities in the domains of neuroscience and artificial intelligence. Japan's commitment to scientific research is evidenced by its significant contributions to this field.

India is in tenth position with a total of 127 articles, which suggests an increase in research activity in this field. India's contributions to the fields of neuroscience and artificial intelligence are evidenced by the number of scientific publications in these areas.

The evidence presented in this table demonstrates that there is a considerable scope for research activity in the domain of artificial intelligence (AI) applications in neuroscience on a global scale. While the United States and China are the foremost countries in this field, numerous countries in Europe and Asia are also making substantial contributions. This illustrates that neuroscience and artificial intelligence research is of global significance and is being actively pursued in numerous countries.

**Figure 8:**
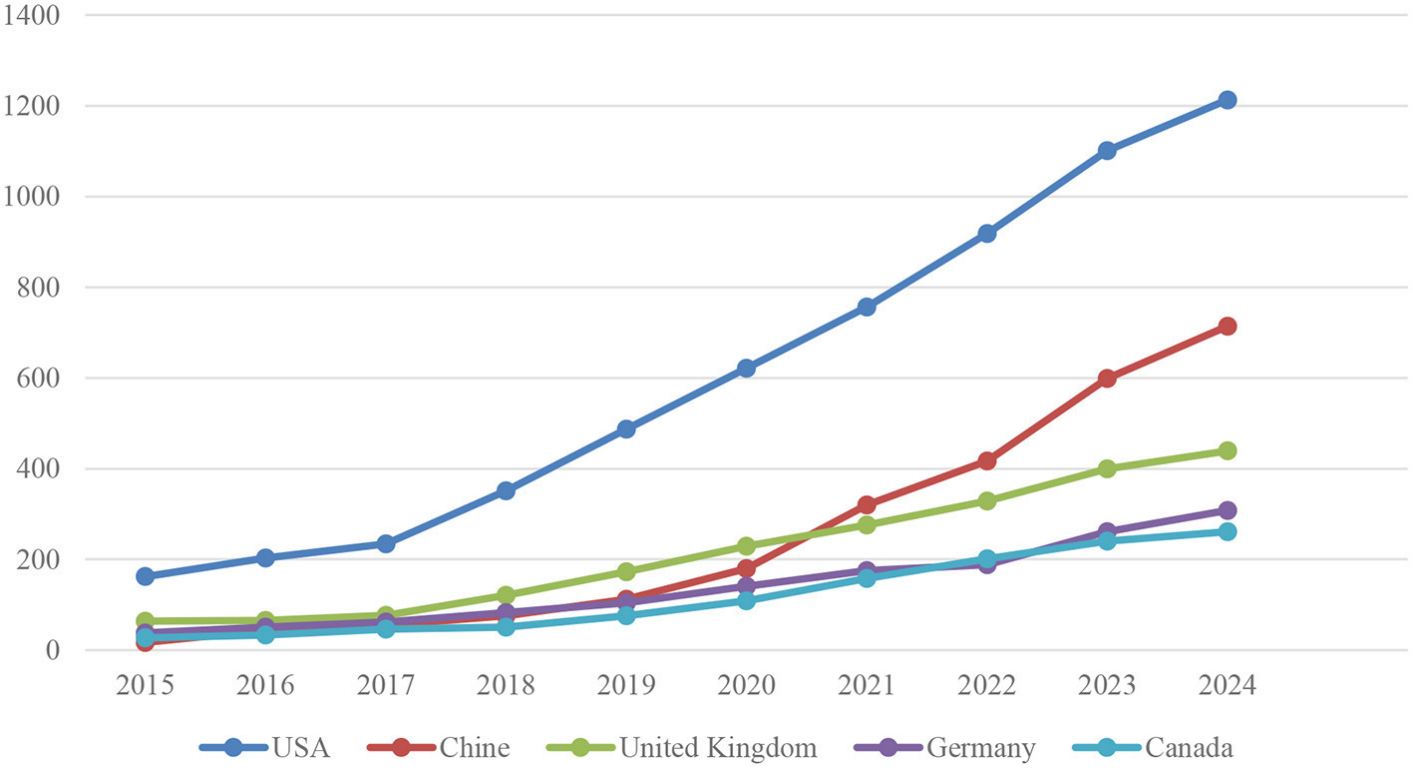
Most relevant country production over time (top 5).

Figure 8 shows the total number of publications by year for the five countries with the highest number of publications in the field of AI applications in neuroscience. This data allows us to assess the growth of research contributions and scientific activity of the respective countries in this field over time.

The United States commenced with 162 articles in 2015 and has demonstrated a consistent growth trajectory, culminating in 1,213 articles in 2024. In particular, there was a significant increase in the number of articles published between 2018 and 2019, with a rise from 351 to 487. This exponential growth demonstrates a significant expansion in the capacity and contribution of US research in this domain. From 2015 to 2024, the number of articles published annually exhibited a consistent upward trajectory.

China has demonstrated a noteworthy expansion in its research output, with an initial output of 17 articles in 2015 and a substantial increase to 714 articles in 2024. In particular, there has been a notable surge in the number of articles published subsequent to 2019. The number of articles increased from 112 to 180 between 2019 and 2020 and from 180 to 320 between 2020 and 2021. This exponential growth is indicative of China's substantial investment in scientific research and its burgeoning interest in neuroscience and artificial intelligence.

The United Kingdom commenced with 64 articles in 2015 and concluded with 439 articles in 2024. There is a notable surge in the number of articles published between 2018 and 2019, with an increase from 121 to 173. This is followed by a consistent rise in the number of articles published from 2019 onwards. This illustrates the UK's robust research output and engagement in this domain.

Germany commenced the period under review with 38 articles and concluded it with 308 articles. The rise in the number of articles from Germany was particularly pronounced between 2018 and 2019, with an increase from 83 to 104. This increase is indicative of Germany's significant role and impact on scientific research in the domains of neuroscience and artificial intelligence.

Canada commenced with 27 articles in 2015 and concluded with 261 articles in 2024. The rise in the number of articles published by Canada was particularly pronounced between 2018 and 2019 (from 51 to 76), with a gradual increase observed from 2019 onwards. This evidence demonstrates that Canada is a significant contributor to research activities in neuroscience and artificial intelligence.

The data presented in this table demonstrate that the five countries with the highest number of publications in the field of artificial intelligence (AI) applications in neuroscience have significantly increased their contributions to this field over time. The United States and China have demonstrated the most rapid and substantial growth. Additionally, the United Kingdom, Germany, and Canada are making notable contributions to this field, exhibiting a consistent and gradual increase. The presented data demonstrate that research in the field of neuroscience and artificial intelligence is experiencing a period of rapid growth on a global scale, with active research being conducted in numerous countries.

Table 5 presents a ranking of the 10 most frequently cited countries in the field of artificial intelligence (AI) applications in neuroscience, along with their respective total number of citations and average number of citations per paper. The data permit an evaluation of the research impact and scientific activity of the respective countries in this field.

**Table 5:** Most cited countries (top 10).

**Country**	**Total count**	**Average article citations**
USA	10,895	38.90
United Kingdom	3,699	33.90
China	2,261	15.60
Canada	1,911	32.40
Switzerland	1,559	103.90
Germany	1,420	20.30
France	637	13.80
Italy	449	8.50
Japan	415	10.40
Spain	391	9.30

The United States is the most frequently cited country in this field, with a total of 10,895 citations, representing an average of 38.90 citations per paper. This evidence demonstrates that research conducted in the United States in the fields of neuroscience and artificial intelligence is of a high standard and has a significant impact. The work of US scientists is frequently cited by their peers.

The United Kingdom occupies the second position with a total of 3,699 citations and an average of 33.90 citations per paper. Additionally, research conducted in the UK is similarly perceived to have a considerable impact and is often referenced. This evidence illustrates the significant role and contribution of the UK in neuroscience and AI research.

China occupies the third position with a total of 2,261 citations, with an average of 15.60 citations per article. Although China has a high total number of citations, its rate of citations per article is comparatively lower than that of other countries. This indicates that China has a substantial research network, yet the number of citations per article is comparatively low.

Canada occupies the fourth position with a total of 1,911 citations and an average of 32.40 citations per paper. The research conducted in Canada has a notable impact on the scientific community, as evidenced by its frequent citation by other researchers. This illustrates the high caliber of scientific work being conducted in Canada with regard to neuroscience and artificial intelligence.

Switzerland is in fifth place with a total of 1,559 citations, with an average of 103.90 citations per paper. This evidence demonstrates that Switzerland's research in this field is of the highest quality and has had a significant impact. Switzerland is markedly superior in terms of the number of citations per article, indicating that its research in this field is widely referenced.

Germany occupies the sixth position with a total of 1,420 citations and an average of 20.30 citations per paper. The research conducted in Germany in the fields of neuroscience and artificial intelligence is frequently cited by other researchers and has a significant impact on the wider academic community.

France is in seventh place with a total of 637 citations, with an average of 13.80 citations per article. France's research is also making a significant impact in this field and is frequently cited by other scientists.

Italy is in eighth place with a total of 449 citations and an average of 8.50 citations per article. While Italy's neuroscience and AI research is less frequently cited by other researchers, it nevertheless constitutes a significant and valuable contribution to the field.

Japan occupies the ninth position with a total of 415 citations, with an average of 10.40 citations per article. Japan's research in this field is frequently cited by other scientists and has a considerable impact on the field.

Spain is in tenth position with a total of 391 citations and an average of 9.30 citations per article. The research conducted in Spain is frequently referenced by other researchers and makes a notable contribution to the field.

Overall, this table shows the research impact and scientific contributions of the most cited countries in the field of AI applications in neuroscience. The United States and the United Kingdom lead in the total number of citations, while Switzerland is the clear leader in the number of citations per paper. China has an extensive research network and ranks third in total citations. Canada and Germany also make significant contributions in this field. These data show that research in neuroscience and artificial intelligence has a significant impact on a global scale and is actively referenced in many countries.

**Figure 9:**
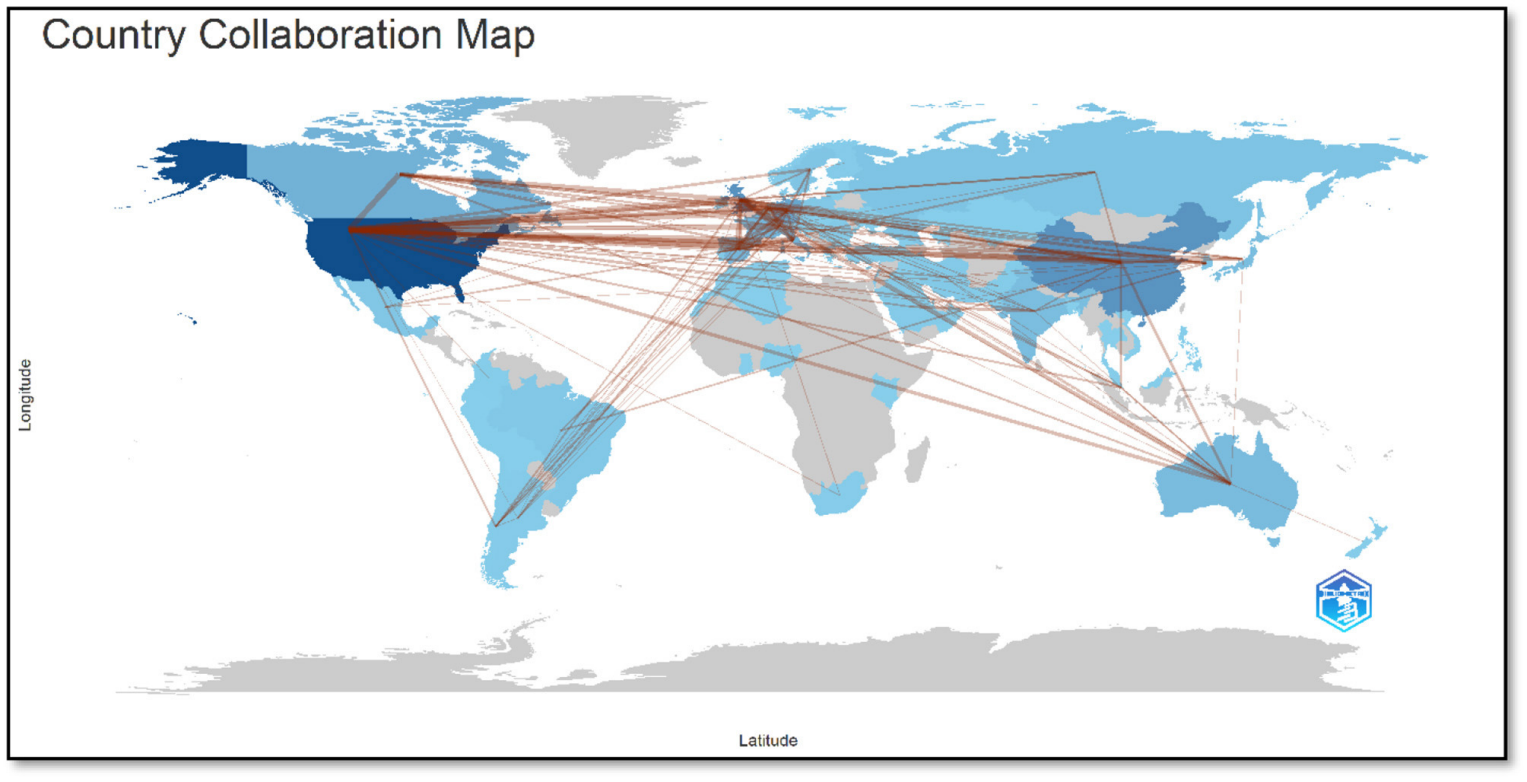
Countries collaboration map.

Figure 9 shows international collaborations in the field of AI applications in neuroscience. The lines on the map represent scientific collaborations between countries, and the density and thickness of the lines reflect the frequency of collaborations.

The United States occupies a central position on the map and is the country that engages in the greatest number of cooperative activities with other countries. The density and thickness of the lines indicate that the United States has particularly robust cooperative relationships with European countries and Canada. The United States has particularly strong cooperative relationships with the United Kingdom, Germany, Canada, and China.

Additionally, the United Kingdom plays a pivotal role in the map, exhibiting robust collaboration with Germany, the United States, Spain, and Australia. Additionally, the United Kingdom maintains robust connections with other nations within the European region.

Germany is noteworthy for its robust collaboration with the United States and the United Kingdom. Additionally, it maintains significant connections with other European nations.

Canada is especially noteworthy for its robust ties with the United States. Furthermore, it engages in collaborative efforts with the United Kingdom.

It has established significant cooperative relationships with the United States and engages in some degree of collaboration with other countries. Australia's robust collaboration with the United Kingdom is a notable feature of the regional landscape. Furthermore, it maintains connections with other nations.

The map illustrates that neuroscience and AI research have a vast network of international collaborations, with a notable concentration between the United States and European countries. This illustrates that scientific research is a global undertaking, and that collaborations between countries facilitate advancements in this field. Table 6 shows the 10 country pairs that collaborate most frequently in the field of AI applications in neuroscience and the frequency of these collaborations.

**Table 6:** Countries collaboration frequency.

**From**	**To**	**Frequency**
USA	United Kingdom	43
USA	Germany	35
USA	Canada	26
USA	China	20
United Kingdom	Germany	19
USA	Italy	15
United Kingdom	Spain	14
USA	Spain	13
United Kingdom	Australia	12
United Kingdom	Canada	12

The United States and the United Kingdom have the highest frequency of collaboration, with 43 instances. Additionally, the United States has collaborated with Germany (35 times), Canada (26 times), and China (20 times), which are also noteworthy. The United Kingdom engages in frequent collaborative endeavors with Germany (19), Spain (14), and Australia (12). Additionally, it is notable that the United States has established significant collaborative relationships with Italy (15), Spain (13), and Canada (12). The data demonstrate that neuroscience and AI research is predominantly concentrated in the United States and the United Kingdom, and that these countries have fostered robust scientific relationships with numerous other nations. These international collaborations play an instrumental role in global developments, facilitating accelerated knowledge sharing and scientific progress.

### 3.4 Document analysis

Table 7 presents the top ten most cited publications in the field of artificial intelligence applications in neuroscience, detailing their sources, total citation counts (TC), average citations per year (TCPY), and references.

**Table 7:** Most cited documents (top 10).

**Paper**	**Source**	**TC**	**TCPY**	**References**
Perceptions of perceptual symbols	Behavioral and Brain Sciences	3,980	153.08	(16)
The social neuroscience of empathy	Annals of the New York Academy of Sciences	946	59.13	(18)
Using goal-driven deep learning models to understand sensory cortex	Nature Neuroscience	740	82.22	(17)
Emotional expressions reconsidered: challenges to inferring emotion from human facial movements	Psychological Science in the Public Interest	738	123.00	(19)
Neuroscience-inspired artificial intelligence	Neuron	715	89.38	(20)
An introduction to deep reinforcement learning	Foundations and Trends in Machine Learning	622	88.86	(21)
A transdisciplinary review of deep learning research and its relevance for water resources scientists	Water Resources Research	527	75.29	(22)
Deep neural networks: a new framework for modeling biological vision and brain information processing	Annual Review of Vision Science	524	52.40	(23)
Bridging biological and artificial neural networks with emerging neuromorphic devices: fundamentals, progress, and Challenges	Advanced Materials	429	71.50	(24)
A deep learning framework for neuroscience	Nature Neuroscience	401	66.83	(25)

TC, total count; TCPY, total count per year; Ref, reference.

“Perceptions of perceptual symbols,” published in Behavioral and Brain Sciences, stands out with a remarkable total of 3,980 citations, averaging 153.08 citations per year, highlighting its profound impact and seminal contribution to the field. Another highly influential paper, “Emotional expressions reconsidered: challenges to inferring emotion from human facial movements,” published in Psychological Science in the Public Interest, averages 123.00 citations per year, underscoring the critical challenges in understanding emotional expressions from facial movements.

Noteworthy contributions include “The Social Neuroscience of Empathy” from Annals of the New York Academy of Sciences, with 946 citations and an annual average of 59.13, illustrating the significant interest in the neural underpinnings of empathy. “Using goal-driven deep learning models to understand sensory cortex” from Nature Neuroscience garners 740 citations with an annual average of 82.22, demonstrating the value of deep learning models in understanding sensory processing. Similarly, “Neuroscience-Inspired Artificial Intelligence” in Neuron, with 715 citations and an average of 89.38 citations per year, bridges the fields of neuroscience and AI, indicating its critical role in advancing AI technologies inspired by brain mechanisms.

The publication “An Introduction to Deep Reinforcement Learning” in Foundations and Trends in Machine Learning has accumulated 622 citations, averaging 88.86 per year, reflecting its foundational impact on the integration of reinforcement learning in neuroscience. “A transdisciplinary review of deep learning research and its relevance for water resources scientists,” published in Water Resources Research, with 527 citations and a yearly average of 75.29, signifies the interdisciplinary reach of deep learning methodologies. “Deep neural networks: a new framework for modeling biological vision and brain information processing” in Annual Review of Vision Science, with 524 citations and an annual average of 52.40, provides a crucial framework for understanding biological vision through neural networks.

Additionally, “Bridging biological and artificial neural networks with emerging neuromorphic devices: fundamentals, progress, and challenges,” published in Advanced Materials, with 429 citations and a yearly average of 71.50, addresses the integration of neuromorphic devices in bridging biological and artificial neural networks. Lastly, “A deep learning framework for neuroscience” in Nature Neuroscience, accumulating 401 citations with an average of 66.83 per year, underscores the importance of deep learning frameworks in advancing neuroscientific research. Collectively, these highly cited publications underscore the interdisciplinary and transformative impact of artificial intelligence and deep learning in the field of neuroscience.

A review of the most frequently cited studies indicates that, over time, the evolution of the integration between neuroscience and AI has been shaped by three main trends. First, the pioneering of basic research is noteworthy. Early studies, particularly those found in works such as Barsalou (16), focused on the foundational conceptual and theoretical underpinnings of neuroscience, contributing to a substantial augmentation in scientific knowledge over time. These foundational studies laid the critical groundwork for a profound and long-term understanding of the relationship between AI and neuroscience, and their long-term impact on the field has been significant.

Secondly, the integration of technology and theory has accelerated, a trend that has gained particular momentum since the mid-2010s. The advent of artificial intelligence has profoundly impacted data analysis and modeling processes within the neuroscience field, particularly through the development of techniques such as deep learning and neural networks. For instance, Yamins and DiCarlo (17) expound on the utilization of deep learning models in elucidating the sensory cortex, underscoring the transformative role of AI as a potent instrument for comprehending biological processes. These developments have enabled neuroscience and AI to advance in a complementary and integrated manner.

The growth of interdisciplinary collaboration has gained significant momentum in recent years. The convergence of neuroscience and artificial intelligence has not only led to technological innovation but also intersected with various disciplines, including the social sciences, ethics, and law, thereby expanding the scope of research. For instance, studies by Singer and Lamn (18) and Barret et al. (19) have integrated neuroscientific perspectives with social and psychological phenomena, addressing topics such as empathy and emotional expressions. Such interdisciplinary approaches have enabled neuroscience research to become more comprehensive, applied, and community-oriented.

The integration of neuroscience and AI will likely explore more areas of innovation, collaboration, and application in the coming years. This diversification of research, ranging from fundamental to applied, aims to maximize the potential of both fields.

### 3.5 Keyword analysis

Table 8 presents the 10 most frequently utilized keywords and their respective frequencies of occurrence within the domain of artificial intelligence (AI) applications in neuroscience.

**Table 8:** Most frequent words (top 10).

**Words**	**Occurrences**
Artificial intelligence	284
Neuroscience	175
Machine learning	88
Deep learning	76
Learning	61
Intelligence	43
Consciousness	39
Computational neuroscience	36
Artificial	31
Cognition	31

The term “artificial intelligence” is the most frequently used, occurring 284 times. This illustrates the pivotal role that AI plays in the field of neuroscience, and the significance of integrating these two disciplines in research endeavors. The second most common term was “neuroscience,” which was used 175 times, underscoring the profound interconnectivity between this field and AI.

The terms “machine learning” (88 times) and “deep learning” (76 times) are also frequently used, indicating that these techniques are widely applied in neuroscience research. The term “learning” is employed on 61 occasions, thereby indicating that the study of general learning processes constitutes an important research topic. Moreover, the terms “intelligence” (43 times) and “consciousness” (39 times) indicate that these two concepts are studied in depth in the fields of AI and neuroscience.

The term “computational neuroscience” is mentioned 36 times, underscoring the significance of computational techniques in neuroscience research. The terms “artificial” and “cognition” are each used 31 times, indicating that artificial systems and cognitive processes play a significant role in the research domain. The recurrence of these keywords underscores the points of convergence between AI and neuroscience and the interdependence of these fields. In sum, the table demonstrates that research in this field is primarily focused on artificial intelligence, learning algorithms, and cognitive processes.

Word cloud is an effective way to visualize the most frequently used words in a given text. The size of the words indicates how often they are used in the text, with larger words representing those used more often and smaller words representing those used less often. This method helps to quickly identify key themes and concepts in a text.

**Figure 10:**
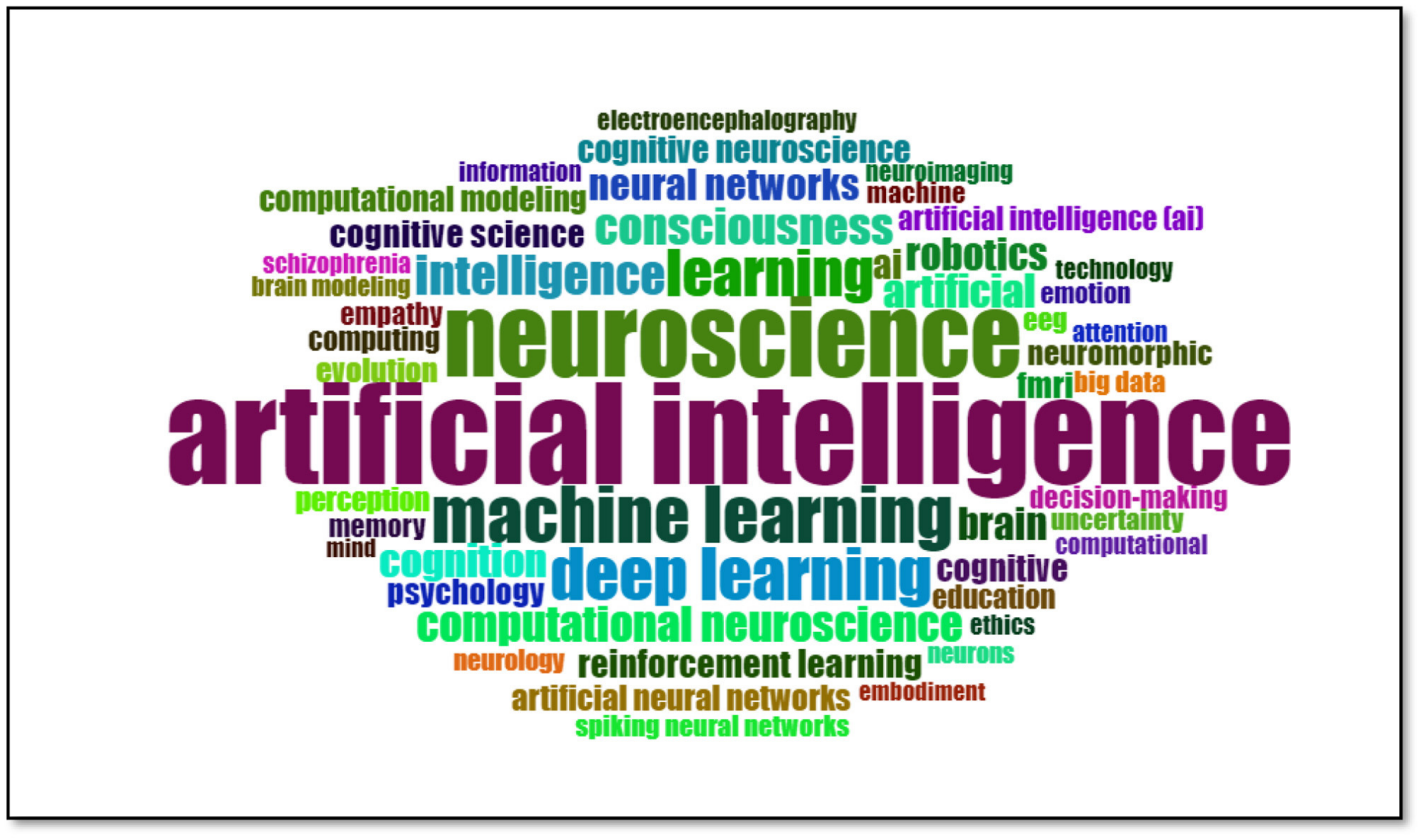
Wordcloud of keywords.

The word cloud presented in Figure 10 shows the most frequently used words in research in neuroscience and artificial intelligence.

The most prominent word in this image is “artificial intelligence,” and its large size makes it evident that artificial intelligence is a central theme in neuroscience studies. Similarly, the prominent display of the word “neuroscience” underscores the primary scientific domain that these studies address.

Other noteworthy terms include “machine learning” and “deep learning,” which refer to techniques commonly utilized in contemporary neuroscience research. The terms “learning” and “intelligence” are also prominent, indicating that a significant portion of the work in this field is dedicated to understanding the processes of learning and intelligence. The terms “consciousness” and “computational neuroscience” underscore the significance of these domains in scientific inquiry.

In addition, the word cloud incorporates terms such as “neural networks,” “cognition,” and “empathy,” which illustrate the diverse facets and subtopics of neuroscience and AI research. In sum, this visualization effectively illustrates the breadth of research at the intersection of neuroscience and AI, as well as the relative prominence of specific concepts within the field. Such an analysis can assist researchers in rapidly acquiring an understanding of the current trends and focal points within the field.

**Figure 11:**
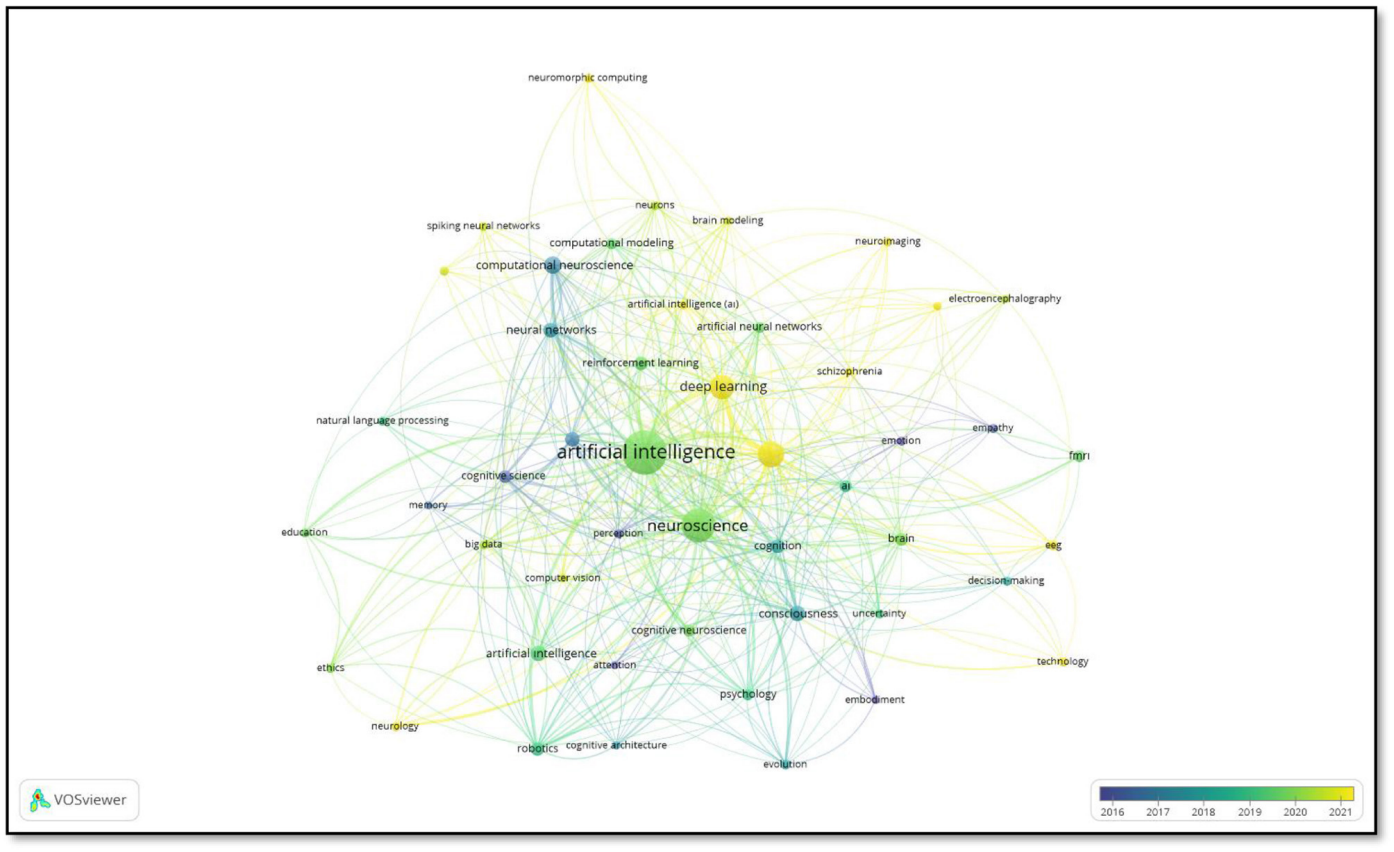
Co-occurrence of keyword.

Figure 11 illustrates the “Co-occurrence of Keywords” analysis, which elucidates the salient research domains in the interdisciplinary fields of artificial intelligence (AI) and neuroscience, as well as the interconnections between these two domains.

The keywords “artificial intelligence” and “neuroscience” occupy a central position in the map, indicating that these two disciplines are in close interaction. In particular, the concepts of “deep learning,” “neural networks,” and “computational neuroscience” reflect the application of AI in neuroscience research. Furthermore, brain imaging techniques, including functional magnetic resonance imaging (fMRI), electroencephalography (EEG), and neuroimaging, play a significant role in AI-supported analyses and are directly linked to neuroscience-related concepts such as cognition, the brain, and decision-making. As indicated by the color scale representing time distribution, while areas such as “cognitive science” and “robotics” gained prominence during the 2016–2017 period, more innovative topics such as “deep learning,” “brain modeling,” and “reinforcement learning” attracted attention during the 2018–2021 period. Furthermore, the presence of the keywords “ethics” and “education” in the network indicates that the ethical and social dimensions of artificial intelligence and neuroscience occupy a significant position within the research domain. These findings demonstrate that artificial intelligence techniques are being increasingly utilized in neuroscience, and interdisciplinary studies are gaining prominence.

**Figure 12:**
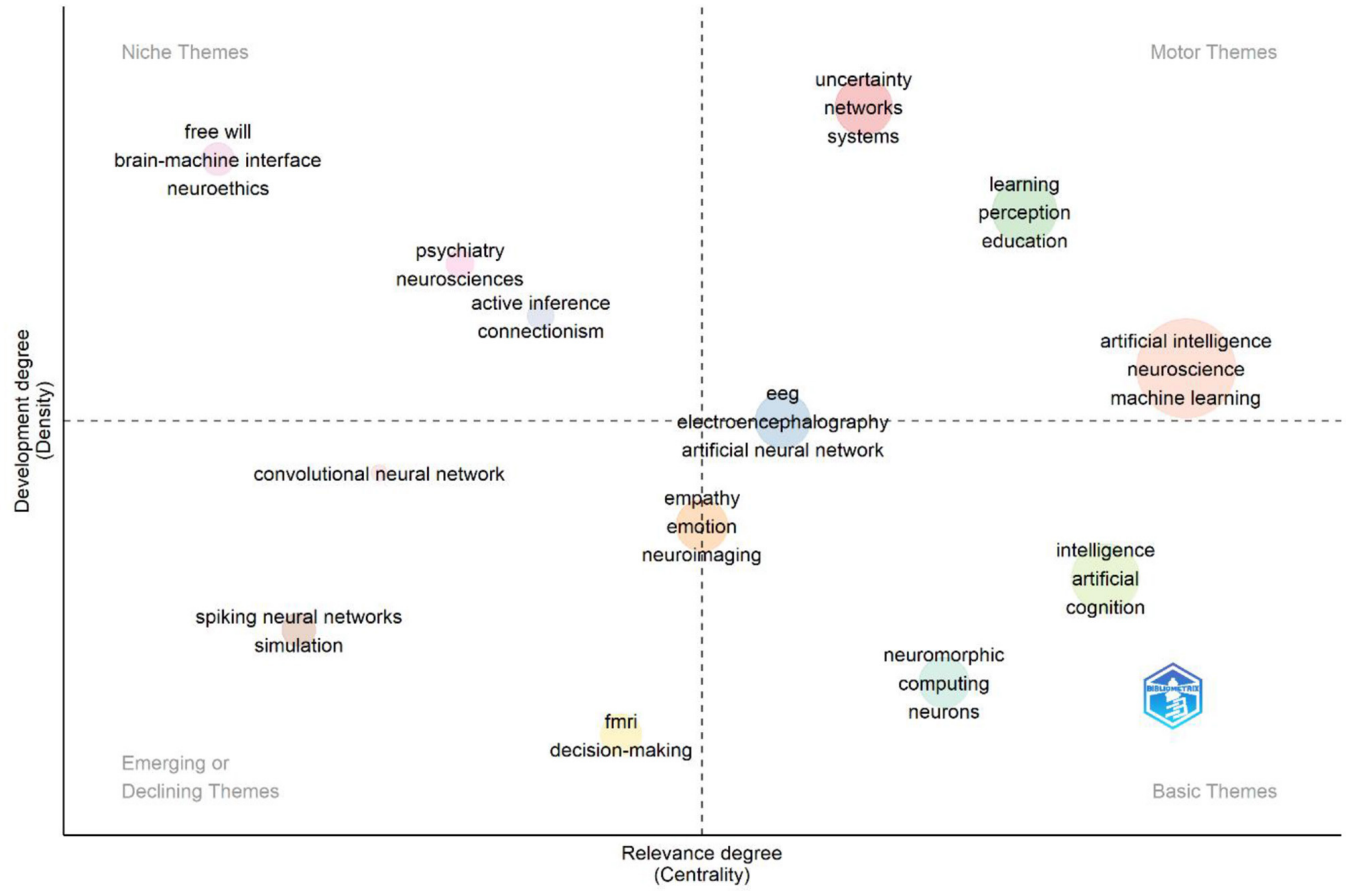
Thematic map of keywords.

The Thematic Map of Keywords is a visualization of keywords divided into four quadrants according to their importance and sophistication. This map shows the prominent themes in a particular research area and how central and developed these themes are. Each quadrant represents different thematic areas and shows the position of specific keywords in these areas. Figure 12 shows the thematic map of keywords used in AI studies in neuroscience.

- Niche themes (top left quadrant)

A niche theme can be defined as a topic that is both high intensity and low centrality, addressing a specific area of expertise. These themes, which have been the subject of extensive study in the literature, have yet to achieve a broad impact. They are of interest to specific research groups. As illustrated in the image, keywords such as “brain-machine interface,” “neuroethics,” and “free will” are particularly prominent. Brain-machine interfaces (BMIs) represent a significant area of research at the nexus of neuroscience and artificial intelligence, as technologies that facilitate individuals' ability to control devices through thought. In contrast, neuroethics addresses the ethical implications of artificial intelligence technologies on neurological data. The theme of “free will” indicates the theoretical debates that have emerged as a result of the intersection of artificial intelligence and brain sciences, which seek to elucidate the nature of human behavior. The niche positioning of these themes indicates that the topics are being discussed in depth within academic circles, but have not yet formed a substantial body of literature.

- Motor themes (top right quadrant)

Motor themes represent areas of research that are both central (high centrality) and advanced (high density) within the existing academic literature. These themes serve as the primary focus of existing scientific work, thereby influencing the direction of the academic literature. As illustrated in the image, the keywords “artificial intelligence,” “neuroscience,” and “machine learning” are particularly prominent among the motor themes. This evidence demonstrates that artificial intelligence and machine learning are employed as a foundational tool in neuroscience research. In particular, machine learning algorithms are known to play a central role in neuroimaging analysis, modeling neural circuits, and understanding brain function. Concurrently, the concepts of “learning,” “perception,” and “education” are among these themes, underscoring the integration of AI-supported learning processes with neuroscience. These findings demonstrate how artificial intelligence has transformed our understanding of perception and learning mechanisms in interdisciplinary studies.

- Emerging or declining themes (lower left quadrant)

These themes are characterized by low centrality and low density, indicating that they represent topics that are either emerging in the literature or have lost interest. The image illustrates key terms such as “spiking neural networks,” “simulation,” and “convolutional neural network” (CNN). Spiking neural networks (SNNs) are a mathematical model of biological neural networks that more accurately reflects the physiological processes of the human brain. Despite the fact that these networks are better able to reflect timing-based information processing than artificial neural networks, they are still not widely adopted in large-scale applications. The theme of “simulation” plays a pivotal role in modeling neurological data and training artificial intelligence systems, yet it has a relatively limited centrality in the extant literature. Although convolutional neural networks (CNNs) are a common technique, particularly in image-based analysis, their inclusion here suggests that the study of CNNs in neuroscience may be approaching a saturation point. This may indicate that, in the future, these fields will adopt more specific or alternative methods.

- Basic themes (lower right quadrant)

Themes of a fundamental nature are of considerable importance, yet they are not yet fully developed. They represent research topics that are significant in the existing literature but have yet to be explored in sufficient depth. These themes encompass areas of academic inquiry that are of significant interest but have yet to be sufficiently elaborated. The image features a number of keywords, including “intelligence,” “artificial,” “cognition,” and “neuromorphic computing.” The field of neuromorphic computing emerged from the convergence of neuroscience and artificial intelligence with the objective of developing more efficient and faster computing systems by emulating the architectural principles of biological nervous systems. This theme is directly linked to developments in the field of cognitive science. Furthermore, the positioning of the themes “intelligence” and “cognition” reflects the efforts of AI systems to comprehend and emulate human intelligence. The basic themes represent potential areas of research that will receive greater attention in future literature, reflecting the ongoing efforts of AI to gain a deeper understanding of cognitive processes.

The strategic map reveals that engine themes (artificial intelligence, machine learning, and neuroscience) demonstrate a robust evolution of applications in neuroscience. Niche themes concentrate on particular subjects, whereas emerging themes are susceptible to potential transformations in the forthcoming years. The basic themes indicate that artificial intelligence and cognitive processes will be critical future research areas. This analysis facilitates comprehension of contemporary trends in the literature and delineates strategic domains of inquiry that researchers may pursue.

## 4 Discussion and future works

This study presents a comprehensive bibliometric analysis of the applications of artificial intelligence in neuroscience. This analysis, which examines a total of 1,208 studies published between 1983 and 2024, demonstrates that research activities pertaining to the application of artificial intelligence in neuroscience have exhibited a gradual yet consistent increase over time. In particular, there has been a notable surge in research activity in this field since the mid-2010s. The integration of AI and neuroscience has resulted in notable advancements in neurological imaging, brain-computer interfaces (BCI), and the diagnosis and treatment of neurological diseases.

The findings from the bibliometric analysis of artificial intelligence (AI) applications in neuroscience elucidate significant insights into the trends, collaborations, and impact of research in this interdisciplinary field. This section will present a synthesis of the key observations derived from the results, integrating the data presented in the tables and figures.

The analysis shows a remarkable growth in publications related to AI in neuroscience, with an annual growth rate of 12.32% from 1983 to 2024. This increasing trend reflects the growing interest and advancements in AI technologies and their applications in understanding and treating neurological conditions. The surge in publications, especially in recent years, underscores the importance of AI in revolutionizing neuroscience research.

The distribution of documents by publication year illustrates this growth, with a notable increase in the number of studies from 2015 onwards. This surge in research activity coincides with significant advances in AI techniques, particularly deep learning, which have demonstrated substantial potential in domains such as neuroimaging and disease diagnosis.

The analysis demonstrates a substantial degree of international collaboration in the domains of artificial intelligence and neuroscience, with an international co-authorship rate of 26.32%. Such collaboration is vital for the advancement of the field, as it facilitates the integration of diverse expertise and perspectives. The mean number of co-authors per document, 3.96, provides further evidence of the collaborative nature of research in this domain.

The geographic distribution of research outputs indicates that the United States is the leading nation in terms of the number of publications, followed by China, the United Kingdom, Germany, and Canada. This distribution serves to illustrate the global interest and investment in the integration of AI with neuroscience. Notable contributors include institutions such as Stanford University, the University of Oxford, and Tsinghua University, which reflect their robust research capabilities and resources in this field.

The average number of citations per document is 21.85, which suggests that research in the fields of artificial intelligence and neuroscience is not only highly productive but also significantly influential. The high citation rates are indicative of the relevance and influence of these studies within the broader scientific community. Notable publications, including those by Chen et al. (10) and Litjens et al. (14), have considerably influenced present-day research trends and methodologies in AI applications for neurodegenerative diseases and neurological diagnostics.

The incorporation of AI in neuroscience is positioned to sustain its accelerated advancement, driven by ongoing technological innovations and interdisciplinary collaborations. Future research should prioritize the resolution of challenges associated with AI applications, including ethical considerations, data privacy, and the interpretability of AI models. Furthermore, extending the remit of AI research to encompass underrepresented regions and cultivating more inclusive collaborations can contribute to the enhancement of the field.

It is imperative to acknowledge the significance of several pivotal avenues for future research. First, ethical and privacy concerns must be accorded a high level of priority in interdisciplinary research initiatives, particularly those that intersect with fields such as artificial intelligence and neuroscience. Of particular importance are the protection of personal data, the ethical boundaries of data use, and the transparency of AI models. A comprehensive examination of these issues is necessary to enhance the credibility of this field.

However, given the complexity and multidimensionality of these research areas, it is imperative to foster interdisciplinary collaboration to develop more innovative and practical solutions. Consequently, it is imperative to convene experts from various disciplines, including neuroscience, artificial intelligence, ethics, and law, to cultivate a more comprehensive and integrated approach to confronting these challenges. In this regard, the establishment of collaborative networks and the implementation of effective communication systems within these networks are of paramount importance.

The development of effective communication strategies, the establishment of multidisciplinary project management, and the cultivation of team building are of paramount importance in this regard. The heterogeneity of research teams has been demonstrated to enhance the success rate of interdisciplinary projects. Nevertheless, meticulous planning of organizational structures and communication protocols is paramount to ensure the effective management of such teams and their harmonious collaboration toward shared objectives.

In conclusion, the bibliometric analysis provides a comprehensive overview of the current state and future potential of AI in neuroscience. The significant growth in publications, collaborative efforts, and impactful research underscore the transformative role of AI in advancing our understanding of the brain and improving neurological healthcare.

## 5 Conclusion

This bibliometric analysis provides a comprehensive evaluation of the research landscape at the intersection of artificial intelligence (AI) and neuroscience. By examining a corpus of 1,208 publications from 1983 to 2024, the study elucidates the significant growth, key research areas, and collaborative nature of this interdisciplinary field. The analysis demonstrates a robust annual growth rate of 12.32% in publications, which underscores the growing significance and practical applications of AI in neuroscience. The data indicates a significant increase in research output, particularly from 2015 onwards, reflecting advancements in AI technologies and their applicability in neurological studies. The primary areas of focus include neuroimaging, brain-computer interfaces (BCIs), and the diagnosis and treatment of neurological diseases. The potential of AI in these areas is evidenced by its high accuracy in disease diagnosis and its capacity to personalize treatment plans, which contribute to improved patient outcomes and a deeper understanding of neurological conditions.

The field is distinguished by a notable degree of international collaboration, as evidenced by a 26.32% rate of international co-authorship. The geographic distribution of research outputs is led by the United States, followed by China, the United Kingdom, Germany, and Canada, indicating a global interest and investment in AI and neuroscience integration. The average citation rate of 21.85 per document serves to illustrate the impact and relevance of research in this field. Notable studies have significantly influenced current methodologies and trends, underscoring the importance of AI in advancing neurological research and healthcare.

The results of this analysis highlight the potential for AI to transform neuroscience. Nevertheless, a number of challenges and opportunities demand further investigation in future research. As artificial intelligence (AI) applications become increasingly integrated into clinical practice, it is of paramount importance to address ethical issues and ensure data privacy. It is imperative that researchers prioritize transparent and ethical practices to maintain public trust and ensure equitable benefits from AI advancements. It is imperative that there be a strengthening of interdisciplinary collaborations between experts in the field of artificial intelligence, neuroscientists, and clinicians, in order to ensure the continued progress and innovation in this field. Collaborative endeavors have the potential to yield more comprehensive and efficacious solutions to complex neurological issues. The expansion of research efforts to include underrepresented regions and populations has the potential to enrich the field and promote more inclusive advancements in AI and neuroscience. The encouragement of diverse perspectives and participation will facilitate a more comprehensive understanding of neurological conditions and their treatments. The ongoing advancement of AI technologies, particularly those pertaining to deep learning and machine learning, will serve to further enhance the capabilities and applications of AI in neuroscience. It is recommended that future research efforts be directed toward the development of more interpretable and robust AI models that can be integrated seamlessly into clinical workflows.

In conclusion, this bibliometric analysis offers valuable insights into the dynamic and rapidly evolving field of artificial intelligence (AI) in neuroscience. The considerable expansion in research output, substantial international collaborations, and high impact of published studies underscore the pivotal role of AI in advancing our understanding and treatment of neurological conditions. As the field continues to mature, it is imperative to address the ethical, collaborative, and technological challenges that lie ahead if we are to realize the full potential of AI in neuroscience. This will ultimately lead to improved neurological healthcare and patient.

## Data Availability

The original contributions presented in the study are included in the article/supplementary material, further inquiries can be directed to the corresponding author.

## References

[1] Esteva A Robicquet A Ramsundar B Kuleshov V DePristo M Chou K . A guide to deep learning in healthcare. Nat Med. (2019) 25:24–9. 10.1038/s41591-018-0316-z 30617335

[2] Aria M Cuccurullo C. Bibliometrix: an R-tool for comprehensive science mapping analysis. J Informetr. (2017) 11:959–75. 10.1016/j.joi.2017.08.007

[3] Reddy A Reddy RP Roghani AK Garcia RI Khemka S Pattoor V . Artificial intelligence in Parkinson's disease: early detection and diagnostic advancements. Ageing Res Rev. (2024) 99:102410. 10.1016/j.arr.2024.102410 38972602

[4] Topol EJ. High-performance medicine: the convergence of human and artificial intelligence. Nat Med. (2019) 25:44–56. 10.1038/s41591-018-0300-7 30617339

[5] Cui J Miao X Yanghao X Qin X. Bibliometric research on the developments of artificial intelligence in radiomics toward nervous system diseases. Front Neurol. (2023) 14:1171167. 10.3389/fneur.2023.1171167 37360350 PMC10288367

[6] Xia X Li L Cheng Z Chen Q Huang T Yu Y . Comprehensive bibliometric research in neuroscience: focusing on ophthalmology. Front Neurosci. (2023) 17:1106023. 10.3389/fnins.2023.1106023 37397445 PMC10308020

[7] Lin C-L Chen Z Jiang X Chen GL Jin P. Roles and research trends of neuroscience on major information systems journal: a bibliometric and content analysis. Front Neurosci. (2022) 16:872532. 10.3389/fnins.2022.872532 35992932 PMC9382099

[8] Wang M Liu X Lai Y Cao W Wu Z Guo X . Application of neuroscience tools in building construction – an interdisciplinary analysis. Front Neurosci. (2022) 16:895666. 10.3389/fnins.2022.895666 35801176 PMC9253515

[9] Ali S Sunanda C Henalatha JN. Epilepsy prediction using machine learning. In: 4th International Conference on Artificial Intelligence and Speech Technology (AIST). Delhi: IEEE (2022), 1–5. 10.1109/AIST55798.2022.10065163

[10] Chen C Engvist O Wang Y Olivecrana M Blaschke T. The rise of deep learning in drug discovery. Drug Discov Today. (2018) 23:1241–50. 10.1016/j.drudis.2018.01.039 29366762

[11] Yeung AWK Goto TK Leung WK. The changing landscape of neuroscience research, 2006–2015: a bibliometric study. Front Neurosci. (2017) 11:120. 10.3389/fnins.2017.00120 28377687 PMC5360093

[12] Duda M Ma R Haber N Wall DP. Use of machine learning for behavioral distinction of autism and ADHD. Transl Psychiatry. (2016) 6:e732. 10.1038/tp.2015.221 26859815 PMC4872425

[13] Aslam N Khan IU Bashamakh A Alghool FA Aboulnour M Alsuwayan NM . Multiple sclerosis diagnosis using machine learning and deep learning: challenges and opportunities. Sensors. (2022) 22:7856. 10.3390/s22207856 36298206 PMC9609137

[14] Litjens G Kooi T Bejnordi BE Setio AAA Ciompi F Ghafoorian M . A survey on deep learning in medical image analysis. Med Image Anal. (2017) 42:60–88. 10.1016/j.media.2017.07.005 28778026

[15] Van Eck NJ Waltman L. Software survey: VOSviewer, a computer program for bibliometric mapping. Scientometrics. (2010) 84:523–38. 10.1007/s11192-009-0146-3 20585380 PMC2883932

[16] Barsalou L. Perceptions of perceptual symbols. Behav Brain Sci. (1999) 22. 10.1017/S0140525X99532147 11301525

[17] Yamins D DiCarlo J. Using goal-driven deep learning models to understand sensory cortex. Nat Neurosci. (2016) 19:356–65. 10.1038/nn.4244 26906502

[18] Singer T Lamn C. The social neuroscience of empathy. Ann N Y Acad Sci. (2009) 1:81–96. 10.1111/j.1749-6632.2009.04418.x 19338504

[19] Barrett LF Adolphs R Pollak SD Marsella S Martinez A. Emotional expressions reconsidered: challenges to inferring emotion from human facial movements. Psychol Sci Public Interest. (2019) 20:68. 10.1177/1529100619832930 31313636 PMC6640856

[20] Hassabis D Kumaran D Summerfield C Botvinick M. Neuroscience-inspired artificial intelligence. Neuron. (2017) 95:245–58. 10.1016/j.neuron.2017.06.011 28728020

[21] François-Lavet V Henderson P Islam R Bellemare M Pineau J. An introduction to deep reinforcement learning. Found Trends Mach Learn. (2018) 11:219–354. 10.1561/2200000071

[22] Shen C. A transdisciplinary review of deep learning research and its relevance for water resources scientists. Water Resour Res. (2017) 54:8558–93. 10.1029/2018WR022643

[23] Kriegeskorte N. Deep neural networks: a new framework for modeling biological vision and brain information processing. Ann Rev Vis Sci. (2015) 1:417–46. 10.1146/annurev-vision-082114-035447 28532370

[24] Tang J Yuan F Shen X Wang Z Rao M He Y . Bridging biological and artificial neural networks with emerging neuromorphic devices: fundamentals, progress, and challenges. Adv Mater. (2019) 31:1902761. 10.1002/adma.201902761 31550405

[25] Richards BA Lillicrap TP Beaudoin P Bengio Y Bogacz R Christensen A . A deep learning framework for neuroscience. Nat Neurosci. (2019) 22:1761–70. 10.1038/s41593-019-0520-2 31659335 PMC7115933

